# Interface Reconstruction of nZVI with a Mesoporous Mg(OH)_2_ Shell: Coupled Mass Transport and Interfacial Electron Transfer During Perchlorate Transformation

**DOI:** 10.3390/nano16211364

**Published:** 2026-10-26

**Authors:** Keke Xu, Yongkang Sun, Lichao Tan, Mingzhu Zhang, Wenming Pei, Ziyan Du, Jiqiang Jia, Xiaoyan Zhang, Jingjing Yun, Feng Ge, Lei Wang, Ningyuan Zhu

**Affiliations:** 1 Nanjing Institute of Environmental Science, Ministry of Ecology and Environment of the People’s Republic of China, Nanjing 210042, China; xukeke@nie.org (K.X.);; 2 College of Environment, Hohai University, Nanjing 210098, China; 3 Xuyi Ecological Environment Comprehensive Administrative Law Enforcement Bureau, Huai’an 211700, China

**Keywords:** perchlorate removal, nZVI@Mg(OH)_2_, core–shell−like structure, interfacial reconstruction, interfacial electron transfer, interfacial impedance

## Abstract

Perchlorate (ClO_4_^−^) is kinetically persistent in aqueous environments because of its highly symmetric tetrahedral structure and the high activation barrier for Cl−O bond cleavage. Although nanoscale zero−valent iron (nZVI) is a strong reductant, particle aggregation, surface passivation, and inefficient interfacial electron transfer limit its reactivity toward ClO_4_^−^. Here, a mesoporous nZVI@Mg(OH)_2_ core–shell−like composite was fabricated by a one−step liquid−phase in situ deposition strategy to couple substrate accessibility with interfacial electron transfer. At a nominal Mg(OH)_2_/nZVI mass ratio of 10%, the Mg(OH)_2_−modified composite showed substantially reduced particle aggregation and increased the BET specific surface area from 4.57 to 67.29 m^2^·g^−1^ and the pore volume from 0.0242 to 0.2542 cm^3^·g^−1^. The hydroxylated Mg(OH)_2_ interface may additionally favor local ClO_4_^−^ accumulation under acidic−to−near−neutral conditions. Under benchmark conditions, the system removed 98.37% of 10 mg·L^−1^ ClO_4_^−^ from the aqueous phase within 48 h. The reaction also showed an apparent activation energy of 50.96 ± 6.04 kJ·mol^−1^. XRD, Raman, and XPS analyses showed progressive Fe^0^ consumption and the development of a Fe_3_O_4_−dominated crystalline reaction layer with a more oxidized and hydroxylated Fe(III)−rich outer region, providing independent evidence for a strongly reducing and dynamically reconstructed Fe−containing interface. Electrochemical measurements revealed a substantially more negative open−circuit potential for nZVI@Mg(OH)_2_, while equivalent−circuit fitting showed that the total polarization resistance decreased from 15.21 to 10.78 kΩ relative to bare nZVI, consistent with a more electrochemically accessible interface. In situ OCP measurements further showed an immediate electrochemical response upon ClO_4_^−^ addition. Together with TBA scavenging and Fe^2+^ control experiments, these results support a predominantly heterogeneous interfacial transformation process and are consistent with a major contribution from direct interfacial electron transfer. The enhanced reactivity is attributed to the coupling of improved substrate accessibility with an electronically accessible, low−resistance Fe−containing interface. This work provides a mechanistic basis for the interfacial design of Fe−based materials with coordinated mass−transport and electron−transfer characteristics toward kinetically persistent oxyanions.

## 1. Introduction

Perchlorate (ClO_4_^−^) is a highly soluble and mobile inorganic oxyanion. It is widely linked to aerospace propellants, fireworks production, and related chemical industries [1]. Although ClO_4_^−^ has a favorable thermodynamic reduction potential, it remains kinetically persistent under typical aqueous conditions. This stability is related to its symmetric tetrahedral structure, delocalized charge, and the high activation barrier for Cl−O bond cleavage [2,3,4,5]. Its high mobility allows ClO_4_^−^ to move from local industrial sources into groundwater and surface water. High ClO_4_^−^ concentrations have been reported in areas affected by intensive fireworks production and related industrial activities [6,7]. Perchlorate exposure is a major health concern because it competitively inhibits iodide uptake by the thyroid. This effect can disrupt thyroid hormone balance. Pregnant women, infants, and children are especially vulnerable during important stages of neurological development [8,9,10,11]. Perchlorate is therefore included in drinking−water management frameworks in several countries and regions. The World Health Organization has set a guideline value of 70 μg·L^−1^ for perchlorate in drinking water [12]. China has set the same limit of 70 μg·L^−1^ under the national Standards for Drinking Water Quality (GB 5749−2022) [13]. Other regions have proposed or adopted lower values. The United States has proposed a health−based value of 20 μg·L^−1^ [14]. Japan has set a target value of 25 μg·L^−1^ [15]. Canada has set a drinking−water screening value of 4 μg·L^−1^ [16].

Current perchlorate treatment methods mainly include physical separation, biological reduction, and chemical reduction. Physical methods such as adsorption and ion exchange can remove ClO_4_^−^ from water, but they mainly transfer the contaminant to another phase. These methods also require further regeneration or disposal of the concentrated waste [2,17,18]. Biological reduction can convert ClO_4_^−^ to chloride. However, its performance depends strongly on microbial activity, electron−donor supply, and environmental conditions [19,20]. Zero−valent iron can directly provide electrons through Fe^0^ oxidation and can reduce ClO_4_^−^ through a relatively simple chemical process. Nanoscale zero−valent iron (nZVI) has a high specific surface area and high surface reactivity. It has therefore been widely studied for perchlorate reduction [4,21,22].

However, the favorable reducing potential of Fe^0^ does not necessarily result in rapid ClO_4_^−^ removal. Bare nZVI can undergo strong magnetic aggregation and nonproductive corrosion. Iron oxide and hydroxide layers also form gradually on its surface. These layers reduce the accessible reactive area and hinder interfacial electron transfer. As a result, ClO_4_^−^ has less access to reactive sites, and electron transport from the Fe^0^ core becomes more difficult [23,24,25,26,27,28]. Surface coatings can reduce particle aggregation and improve particle stability. However, organic coatings may degrade or detach in complex water systems. Dense insulating coatings can also hinder electron transfer across the interface [27,28,29]. An attractive alternative is to construct a functional inorganic shell that regulates the Fe^0^/water interface rather than merely isolating the iron core. Mg(OH)_2_ is a weakly alkaline, hydroxyl−rich inorganic phase with favorable environmental compatibility and has previously been incorporated into nZVI−based materials. Mg(OH)_2_−containing nZVI composites have been reported to suppress nanoparticle aggregation, improve suspension stability and mobility, protect Fe^0^ from rapid aqueous corrosion, and prolong the release of reductive reactivity in systems targeting Pb(II) [30], Cr(VI) [31,32], U(VI) [31,33], and related contaminants. In particular, Hu et al. demonstrated that encapsulating nZVI within a soluble Mg(OH)_2_ shell markedly reduced magnetic aggregation, improved transport through saturated porous media, protected the Fe^0^ core during storage and transport, and enabled controlled release of nZVI reactivity as the shell dissolved [34]. Maamoun et al. further showed that Mg(OH)_2_ coating suppressed rapid Fe^0^ corrosion and maintained long−term reductive reactivity toward Cr(VI) over extended reaction periods [32]. These observations suggest that Mg(OH)_2_ can function as an interfacial regulator rather than as a passive coating. However, this concept has not been systematically resolved for the kinetically recalcitrant ClO_4_^−^ reduction reaction. It remains unclear whether a Mg(OH)_2_ shell can keep ClO_4_^−^ transport pathways open, regulate the local interface, and improve electron transport across the changing Fe^0^/oxide/water interface. More importantly, the relationship among shell structure, interfacial charge−transfer resistance, Fe−phase transformation, and the relative contributions of different electron−transfer pathways during perchlorate transformation remains insufficiently understood. The potential electrostatic contribution of the hydroxylated Mg(OH)_2_ interface to local ClO_4_^−^ enrichment also requires mechanistic evaluation rather than assumption.

To address these knowledge gaps, an nZVI@Mg(OH)_2_ core–shell composite was constructed in this study through a one−step liquid−phase in situ deposition strategy. We hypothesized that an appropriately structured Mg(OH)_2_ shell could couple substrate accessibility with electron accessibility: the porous shell would maintain mass−transfer pathways toward the Fe−containing reaction interface, while regulation of aggregation, surface passivation, and local interfacial chemistry would preserve efficient electron delivery from the Fe^0^ core. This hypothesis was examined by (i) resolving the core–shell−like morphology, pore structure, crystalline phases, and elemental chemical states; (ii) quantifying perchlorate−removal kinetics under variations in material dosage, pollutant loading, pH, temperature, and coexisting anions; and (iii) integrating activation−energy analysis, in situ electrochemical measurements, solid−phase evolution, and mechanistic control experiments to evaluate the contribution of heterogeneous interfacial electron−transfer process. Particular attention was given to whether the reconstructed interface could promote local ClO_4_^−^ accessibility while reducing charge−transfer resistance, thereby establishing a mechanistic basis for coordinated mass and electron transport during perchlorate treatment.

## 2. Materials and Methods

### 2.1. Chemicals and Reagents

All chemicals were of analytical grade and were used without further purification. Ferric chloride hexahydrate (FeCl_3_·6H_2_O, 99%), potassium perchlorate (KClO_4_, 99%) and ferrous sulfate monohydrate (FeSO_4_·H_2_O, 98%) were purchased from Shanghai Macklin Biochemical Technology Co., Ltd., Shanghai, China. FeCl_3_·6H_2_O was the Fe(III) precursor used throughout the synthesis experiments. Magnesium hydroxide (Mg(OH)_2_, 95%) and tert−butanol (TBA, 99.5%) were purchased from Shanghai Aladdin Biochemical Technology Co., Ltd., Shanghai, China. Sodium borohydride (NaBH_4_, 98%), sodium chloride (NaCl, ≥99.5%), sodium nitrate (NaNO_3_, 99%), and sodium sulfate (Na_2_SO_4_, 99%) were used as received. HCl and NaOH solutions (0.1 mol·L^−1^) were used to adjust the initial pH. More details on the chemicals and solution preparation are provided in Texts S1 and S2.

### 2.2. Synthesis of Bare nZVI and nZVI@Mg(OH)_2_

Bare nZVI and nZVI@Mg(OH)_2_ were synthesized by liquid−phase reduction under an N_2_ atmosphere [26]. For bare nZVI synthesis, 4.889 g of FeCl_3_·6H_2_O (18.09 mmol Fe(III)) was dissolved in 20 mL of deionized water and then mixed with 80 mL of absolute ethanol. The mixture was mechanically stirred for 1 h under continuous high−purity N_2_ protection. A freshly prepared NaBH_4_ solution containing 2.735 g of NaBH_4_ (72.29 mmol) in 100 mL of deoxygenated deionized water was then added dropwise at 5 mL·min^−1^ under vigorous stirring. After complete addition, the suspension was further stirred for 1 h to allow sufficient reduction of Fe^3+^ to Fe^0^. The resulting black solids were collected by filtration. The solids were washed three times with alternating deoxygenated deionized water and absolute ethanol. They were then vacuum−dried at 50 °C for 24 h, and stored in an N_2_−filled glovebox until use.

The nZVI@Mg(OH)_2_ composites were prepared using the same reduction procedure. A predetermined amount of Mg(OH)_2_ was first dispersed in 80 mL of absolute ethanol. The suspension was ultrasonicated for 10 min and then mixed with the FeCl_3_ precursor solution. For the nominal 10% formulation, 0.105 g of Mg(OH)_2_ (1.80 mmol) was added to the Fe(III) precursor solution containing 4.889 g of FeCl_3_·6H_2_O (18.09 mmol Fe), corresponding to approximately 1.01 g of theoretical Fe^0^ and an Mg(OH)_2_/nZVI feed mass ratio of approximately 10.4%. The Mg(OH)_2_ dosage was similarly adjusted to prepare composites with nominal Mg(OH)_2_/nZVI feed mass ratios of 1%, 10%, and 20% for the coating−ratio screening experiments. For convenience, these composites are hereafter denoted as nZVI@Mg(OH)_2_−1, nZVI@Mg(OH)_2_−10, and nZVI@Mg(OH)_2_−20, respectively. After the Fe precursor and Mg(OH)_2_ dispersion had been mixed under N_2_ for 1 h, the freshly prepared NaBH_4_ solution was introduced at 5 mL·min^−1^, followed by an additional 1 h of stirring. The products were filtered, washed, vacuum−dried, and stored under N_2_ following the same procedure as that used for bare nZVI.

### 2.3. Material Characterization and Electrochemical Measurements

The morphology, elemental distribution, crystalline structure, surface chemical states, functional groups, and pore properties of the materials were systematically characterized. SEM coupled with energy−dispersive X−ray spectroscopy (SEM−EDS) was used to examine particle morphology and elemental distribution. Transmission electron microscopy (TEM and HRTEM) was used to resolve the nanoscale structure and lattice fringes. High−angle annular dark−field scanning transmission electron microscopy (HAADF−STEM) coupled with EDS line−scan analysis was further employed to examine the spatial distributions of Fe and Mg across selected particle regions. X−ray diffraction (XRD), X−ray photoelectron spectroscopy (XPS), Fourier−transform infrared spectroscopy (FTIR), Raman spectroscopy, and N_2_ adsorption–desorption analysis were used to characterize the crystalline phases, surface chemical states, functional groups, and textural properties, respectively. XPS binding energies were calibrated using the C 1s peak at 284.8 eV. Peak deconvolution was performed using Thermo Scientific Avantage software (version 6.8.0). Detailed instrumental parameters and sample−preparation procedures are provided in Text S3.

Electrochemical measurements were conducted in a conventional three−electrode configuration using 0.1 M Na_2_SO_4_ as the electrolyte. An Ag/AgCl electrode and a Pt wire served as the reference and counter electrodes, respectively. The catalyst ink was prepared by dispersing 10.0 mg of material and 15 μL of 5 wt% Nafion solution in 1.0 mL of isopropanol and 0.5 mL of ultrapure water. The suspension was ultrasonicated for 30 min to obtain a homogeneous ink. A 15 μL aliquot of the ink was then drop−cast onto a polished glassy carbon electrode (GCE, 3 mm in diameter). The modified electrode was vacuum−dried at 60 °C for 8 h before use.

Cyclic voltammetry (CV) was performed over a potential range of −1.0 to +1.0 V at a scan rate of 50 mV·s^−1^. Electrochemical impedance spectroscopy (EIS) was performed using a CHI660E electrochemical workstation (Shanghai Chenhua Instrument Co., Ltd., Shanghai, China) at the stabilized open−circuit potential of each electrode over a frequency range of 10^5^–10^−1^ Hz with an AC perturbation amplitude of 5 mV. The impedance spectra were fitted using equivalent electrical circuits containing the solution resistance (*R_s_*), interfacial resistance elements (*R_i_*), and constant−phase elements (CPEs) to account for non−ideal capacitive behavior of the heterogeneous Fe−containing interfaces, as commonly considered in electrochemical analyses of ZVI systems [35]. Bare nZVI was adequately described by *R_s_* − (*R*_1_//*CPE*_1_), whereas the nZVI@Mg(OH)_2_ spectrum required two relaxation processes and was fitted using *R*_s_ − (*R*_1_//CPE_1_) − (*R*_2_//*CPE*_2_). Because the modified electrode exhibited two resolvable interfacial relaxation processes, the individual resistance terms were reported separately and their sum was used as the total polarization resistance (*R*_p_) for comparison between the two materials. Fitting quality was evaluated using the chi−squared (χ^2^) value and parameter errors.

For in situ open−circuit potential (OCP) measurements, the electrode was first equilibrated in blank electrolyte for approximately 600 s. A concentrated perchlorate solution was then injected to obtain a final ClO_4_^−^ concentration of 10 mg·L^−1^, and the OCP response was continuously monitored for 1200 s.

### 2.4. Batch Perchlorate Removal and Mechanistic Control Experiments

Batch perchlorate−removal experiments were performed in 23 mL amber glass vials sealed with PTFE−lined septa. The reactors were completely filled to eliminate headspace and were agitated in the dark at 300 rpm in a thermostatic shaker. To avoid changes in liquid volume, solid−to−liquid ratio, and oxygen exposure caused by repeated sampling, a sacrificial sampling protocol was employed, with independent reactors assigned to individual sampling times. At predetermined time points during the 48 h reaction period, the corresponding reactors were opened. The suspensions were immediately filtered through 0.22 μm polyethersulfone (PES) membranes to terminate the reaction. The filtrates were refrigerated before chemical analysis. Blank controls without reactive material were conducted in parallel.

Unless otherwise specified, the reference conditions were an initial ClO_4_^−^ concentration of 10 mg·L^−1^, an initial pH of 4.0, and a reaction temperature of 40 °C. Each reactor contained 0.10 g of nZVI@Mg(OH)_2_ corresponding to a material dosage of approximately 4.35 g·L^−1^. The effect of Mg(OH)_2_ loading ratio was evaluated using bare nZVI and nZVI@Mg(OH)_2_−1, nZVI@Mg(OH)_2_−10, and nZVI@Mg(OH)_2_−20. Catalyst dosages of 0.05, 0.075, 0.10, 0.125, and 0.15 g were tested. The effect of initial pH was evaluated at nominal pH values of 2.0, 4.0, 6.0, 8.0, and 10.0 using 0.1 mol·L^−1^ HCl or NaOH. The corresponding measured initial pH values were 1.80, 4.00, 6.12, 7.88, and 9.96, respectively. To resolve the evolution of solution chemistry during reaction, pH was additionally monitored at 0, 0.5, 1, 2, 3, 6, 12, 24, 36, and 48 h for each initial−pH condition. Temperature effects were investigated at 20, 30, 40, and 50 °C. Initial ClO_4_^−^ concentrations of 5, 10, 50, and 100 mg·L^−1^ were tested to evaluate the effect of pollutant loading. The effects of coexisting anions were examined by separately adding Cl^−^, NO_3_^−^, or SO_4_^2−^ at final concentrations of 100, 200, 500, and 1000 mg·L^−1^. NaCl, NaNO_3_, and Na_2_SO_4_ were used as the corresponding anion sources.

To probe the possible contributions of TBA−quenchable reactive species to ClO_4_^−^ transformation, TBA was introduced at concentrations of 0, 50, 100, 150, and 200 mM. In parallel, Fe^2+^ was tested at 10 mg·L^−1^ either alone or together with nZVI@Mg(OH)_2_ to evaluate the possible contribution of homogeneous Fe^2+^−mediated reduction. These control experiments were used in combination with the electrochemical measurements to evaluate the relative importance of heterogeneous interfacial electron−transfer processes.

### 2.5. Analytical Methods and Kinetic Analysis

Residual ClO_4_^−^ concentrations were measured by ion chromatography using a Dionex ICS−600 system (Thermo Fisher Scientific, Waltham, MA, USA). The system was equipped with an IonPac AS19 analytical column (4 × 250 mm) and a corresponding guard column. KOH was used as the eluent under gradient conditions at a flow rate of 1.0 mL·min^−1^. The injection volume was 250 μL, and the suppressor current was set to 120 mA. All samples were filtered through 0.22 μm membranes before analysis. Quantification was performed using external calibration, with calibration coefficients of determination (R^2^) ≥ 0.999 and relative standard deviations below 5% for parallel measurements.

The temporal evolution of ClO_4_^−^ concentration was fitted using a pseudo−first−order kinetic model:

(1) $$\ln\;(\frac{C_{0}}{C_{t}}{)\;=\;k}_{\mathrm{obs}}t$$

where *C*_0_ and *C*_t_ are the ClO_4_^−^ concentrations at the initial time and at reaction time t, respectively, and *k_obs_* is treated here as an apparent removal rate constant. It does not, by itself, quantify the fraction of ClO_4_^−^ converted to specific chlorine−containing reduction products because the kinetic analysis was based on the decrease in aqueous ClO_4_^−^ concentration.

The corresponding reaction half−life was calculated as:

(2) $$t_{1/2}\;=\frac{\ln2}{k_{\mathrm{obs}}}$$

The apparent activation energy was determined using the linearized Arrhenius equation:

(3) $${\mathrm{lnk}}_{\mathrm{obs}}=\ln A-\;\frac{E_{a}}{RT}$$

where *A* is the pre−exponential factor (h^−1^), *E*_a_ is the apparent activation energy (kJ·mol^−1^), *R* is the universal gas constant (8.314 J·mol^−1^·K^−1^), and T is the absolute temperature (K).

Each set of single−factor experiments was conducted independently using a separately synthesized batch of nZVI@Mg(OH)_2_, and kinetic comparisons were therefore made within each experimental series. Each set of single−factor experiments was conducted independently using a separately synthesized batch of nZVI@Mg(OH)_2_, and kinetic comparisons were therefore made within each experimental series. All batch experiments were performed in triplicate (*n* = 3), and the results are reported as arithmetic means ± standard deviations (SD).

## 3. Results and Discussion

### 3.1. Structural and Morphological Characterization

**Figure 1:**
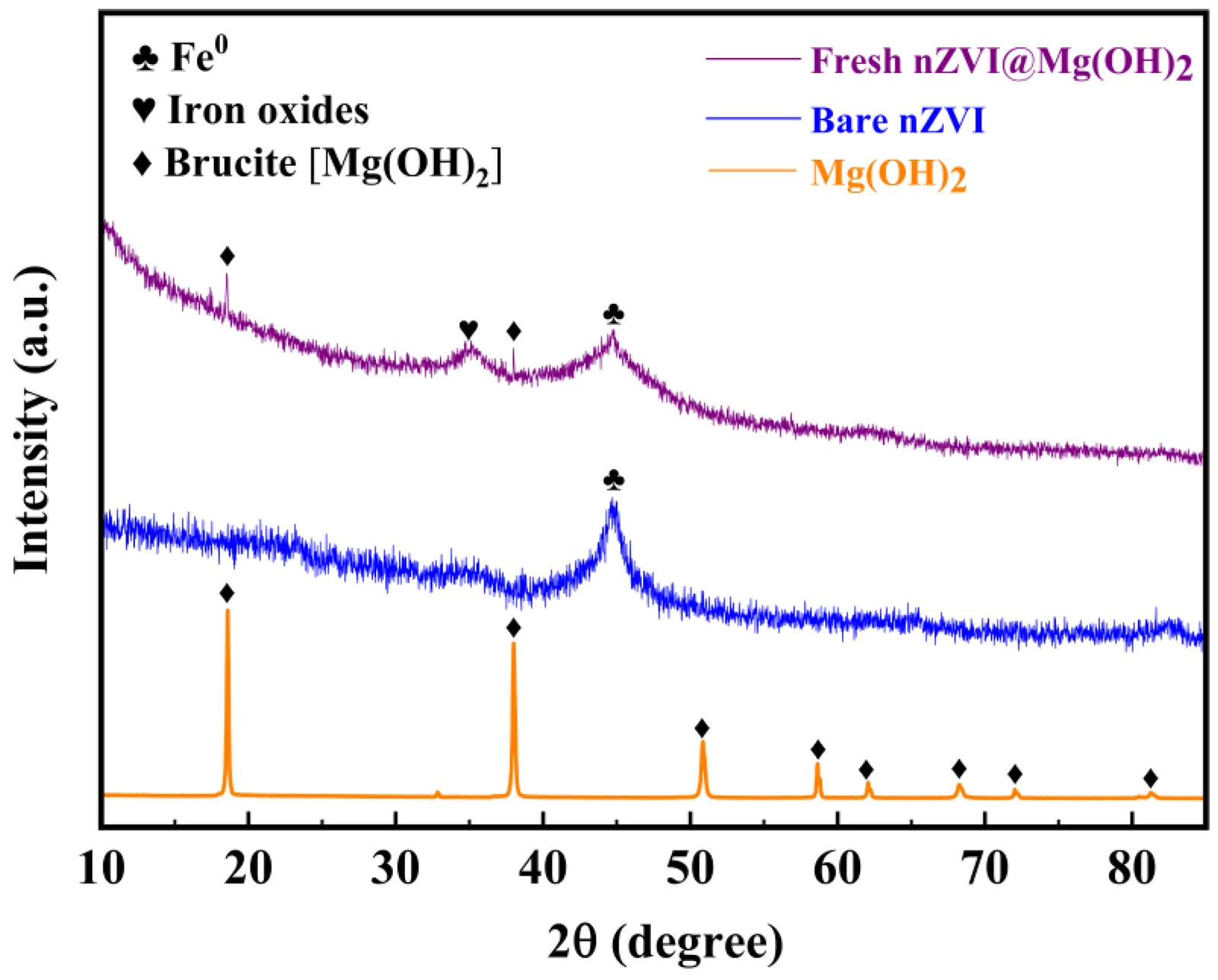
XRD patterns of pristine Mg(OH)_2_, bare nZVI, and fresh nZVI@Mg(OH)_2_. The characteristic reflection positions of α−Fe^0^, Fe−containing oxides, and brucite−type Mg(OH)_2_ are indicated by ♣, ♥, and ♦, respectively.

The phase composition, morphology, interfacial structure, and textural properties of bare nZVI and nZVI@Mg(OH)_2_−10 were systematically characterized to elucidate the structural reconstruction induced by the Mg(OH)_2_ modification. XRD patterns of pristine Mg(OH)_2_, bare nZVI, and fresh nZVI@Mg(OH)_2_ are shown in Figure 1. To improve the resolution of the weak diffraction features in the composite, the XRD pattern of fresh nZVI@Mg(OH)_2_ was collected at a scan rate of 1 °·min^−1^. Bare nZVI exhibited the dominant diffraction reflection of body−centered−cubic α−Fe^0^ at approximately 2θ = 44.7°, corresponding to the (110) plane. Much weaker α−Fe^0^ reflections were observed in the higher−angle region. A broad feature centered around 35° was also present and is attributed more cautiously to Fe−containing oxide phases associated with partial surface oxidation of nZVI.

After Mg(OH)_2_ modification, the dominant α−Fe^0^(110) reflection remained clearly detectable, indicating preservation of the crystalline Fe^0^ phase. In addition, weak diffraction features appeared near 2θ = 18.5° and 38.0°. These positions coincide with characteristic reflections of the pristine brucite−type Mg(OH)_2_ reference. However, because the Mg(OH)_2_−related features in the composite remain weak and the region around 35–38° may also contain contributions from poorly crystalline Fe−containing oxides, these reflections are not regarded as unambiguous evidence for Mg(OH)_2_ by XRD alone.

**Figure 2:**
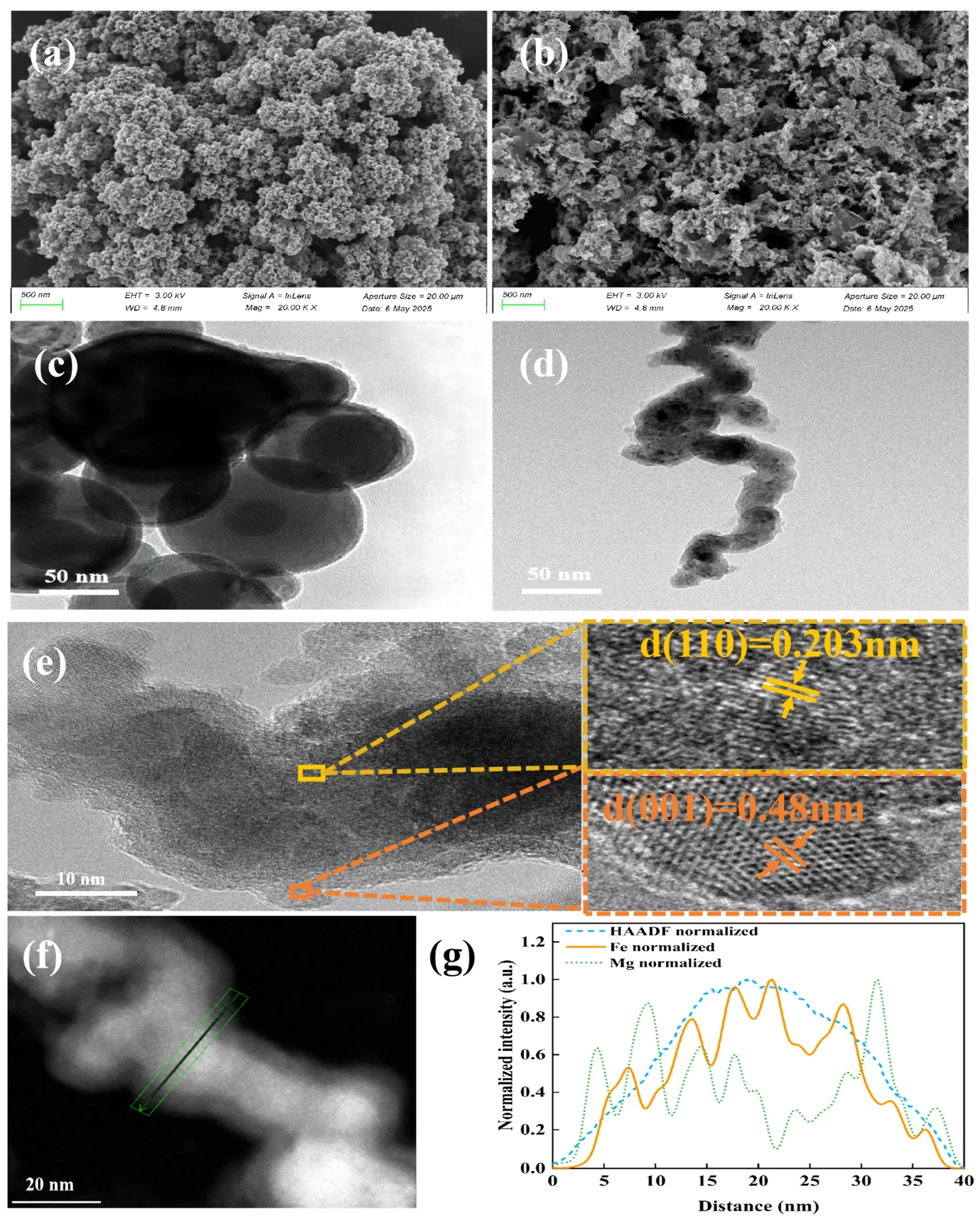
Morphological and interfacial characterization of bare nZVI and nZVI@Mg(OH)_2_−10. SEM images of (**a**) bare nZVI and (**b**) nZVI@Mg(OH)_2_−10 acquired at the same magnification; TEM images of (**c**) bare nZVI and (**d**) nZVI@Mg(OH)_2_−10; (**e**) HRTEM image of nZVI@Mg(OH)_2_−10 with enlarged lattice regions assigned to α−Fe^0^(110) and Mg(OH)_2_(001); (**f**) HAADF–STEM image showing the selected EDS line−scan path across nZVI@Mg(OH)_2_; and (**g**) corresponding normalized HAADF, Fe, and Mg intensity profiles along the scan direction. The colored dashed boxes and connecting lines in (**e**) indicate the regions selected for enlarged HRTEM views, with the corresponding lattice fringes marked by parallel lines and arrows. The green outlined region in (**f**) indicates the STEM–EDS line−scan path.

The SEM and TEM images in Figure 2 clearly show the morphological differences between bare nZVI and nZVI@Mg(OH)_2_−10. At the same SEM magnification, bare nZVI exhibited relatively dense and compact agglomerates (Figure 2a), whereas nZVI@Mg(OH)_2_−10 showed a more open and dispersed particle architecture with less pronounced large−scale aggregation (Figure 2b). These features show that the Mg(OH)_2_ coating effectively improved particle dispersion. TEM observations further revealed distinct nanoscale morphologies. Bare nZVI consisted predominantly of closely associated, electron−dense particles (Figure 2c), whereas nZVI@Mg(OH)_2_−10 exhibited smaller Fe−containing domains embedded within a less compact surrounding structure (Figure 2d). Elemental mapping further confirmed the coexistence and spatial association of Fe, Mg, and O within the composite (Figure S1). HRTEM analysis of nZVI@Mg(OH)_2_−10 revealed lattice spacings of approximately 0.203 and 0.48 nm, which were assigned to the α−Fe^0^(110) and brucite−type Mg(OH)_2_(001) planes, respectively (Figure 2e). The spatial proximity of these lattice domains indicates intimate contact between the Fe−containing region and the Mg(OH)_2_−containing phase. To further resolve their spatial relationship, HAADF−STEM/EDS line−scan analysis was performed across a representative particle region (Figure 2f,g). The Fe signal was predominantly associated with the interior portion of the particle region, whereas Mg exhibited relatively enhanced signals toward the outer portions of the scan. This spatial distribution, together with the HRTEM observations, is consistent with an Mg−containing outer region surrounding an Fe−rich interior. Collectively, the SEM, TEM, HRTEM, and line−scan results support the formation of an Mg(OH)_2_−coated, core–shell−like architecture rather than a compact, uniformly continuous shell.

**Figure 3:**
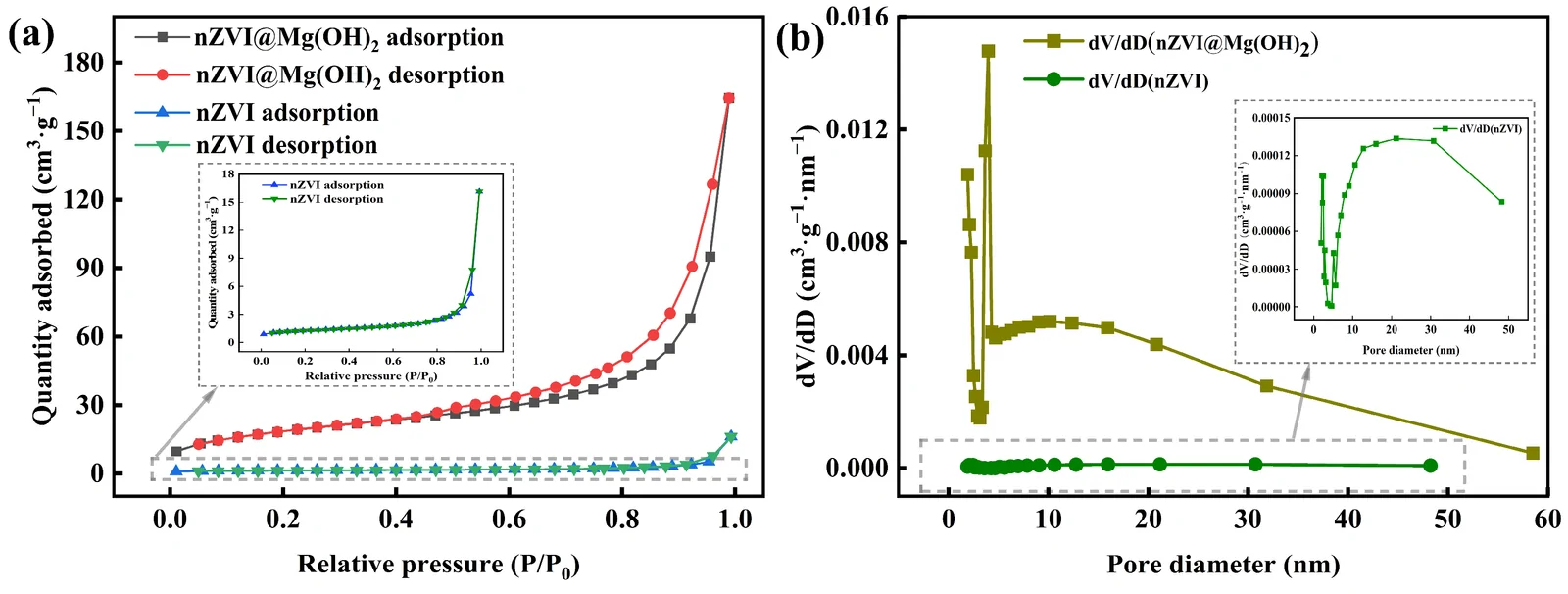
(**a**) N_2_ adsorption–desorption isotherms of nZVI and nZVI@Mg(OH)_2_−10 (the inset shows the enlarged view of the isotherm for nZVI); (**b**) pore size distribution curves of nZVI and nZVI@Mg(OH)_2_−10. The gray dashed rectangles highlight the regions shown in the insets, while the gray arrows indicate the corresponding magnified views.

Mg(OH)_2_ modification also markedly reconstructed the textural properties of the material (Figure 3). Bare nZVI exhibited an N_2_ adsorption–desorption isotherm predominantly resembling Type II behavior, with low N_2_ uptake over most of the relative−pressure range followed by a pronounced increase as P/P_0_ approached unity. Together with its low BET specific surface area of 4.57 m^2^·g^−1^ and total pore volume of 0.0242 cm^3^·g^−1^, this result shows that bare nZVI has limited intrinsic porosity. Most of its accessible pore volume therefore originates from textural voids formed by disordered particle packing. In contrast, nZVI@Mg(OH)_2_−10 exhibited a Type IV isotherm with a pronounced H3−type hysteresis loop at P/P_0_ = 0.5–0.9. This behavior demonstrates the development of substantial textural mesoporosity within the reconstructed shell and interparticle structure. The BET specific surface area increased by approximately 14.7−fold, from 4.57 to 67.29 m^2^·g^−1^, while the total pore volume increased by more than one order of magnitude, from 0.0242 to 0.2542 cm^3^·g^−1^ (Table S1). The BJH pore−size distribution (Figure 3b) showed a dominant mesoporous population centered at approximately 4 nm. A broader secondary distribution was also observed between 5 and 30 nm. These features confirm the formation of a hierarchical mesoporous structure after Mg(OH)_2_ modification.

Collectively, these results demonstrate that Mg(OH)_2_ modification transformed strongly aggregated nZVI into a core–shell−like architecture with markedly enhanced particle dispersion, accessible surface area, and textural mesoporosity. The porous Mg(OH)_2_ shell suppresses magnetic aggregation while providing abundant transport channels and an enlarged solid–liquid interfacial area, thereby facilitating the accessibility of ClO_4_^−^ to Fe−containing reaction interfaces. This structural reconstruction establishes the physical framework for the enhanced perchlorate reduction and interfacial electron−transfer behavior demonstrated in the following sections.

### 3.2. Perchlorate Removal Performance and Apparent Kinetic Response

#### 3.2.1. Effects of Mg(OH)_2_ Loading, Material Dosage, and Initial Perchlorate Concentration

The Mg(OH)_2_ loading was first optimized under the benchmark reaction conditions. As shown in Figure S2, bare nZVI exhibited very limited reactivity toward ClO_4_^−^, whereas Mg(OH)_2_ modification markedly enhanced perchlorate removal. Among the tested formulations, nZVI@Mg(OH)_2_−10, corresponding to a nominal Mg(OH)_2_/nZVI feed mass ratio of 10%, achieved the highest removal efficiency of 98.37% after 48 h and was therefore selected for all subsequent experiments. Hereafter, nZVI@Mg(OH)_2_ refers to the nominal 10% formulation unless otherwise specified. The pronounced difference between bare and coated nZVI indicates that the Mg(OH)_2_ shell does not simply act as an inert surface layer but substantially alters the accessibility and reactivity of the Fe−containing interface.

To examine whether this enhancement could be explained primarily by the increase in BET specific surface area, a high−surface−area B, N−doped porous−carbon−supported nZVI reference was also examined. Although this reference exhibited a BET surface area comparable to that of nZVI@Mg(OH)_2_ (65.37 vs. 67.29 m^2^·g^−1^), its 48 h ClO_4_^−^ removal was substantially lower (59.91% vs. 98.37%; Table S2). This comparison indicates that increased surface area alone is insufficient to account for the enhanced performance of nZVI@Mg(OH)_2_. Rather, the improvement is consistent with the combined effects of structural accessibility and the altered interfacial properties of the Mg(OH)_2_−modified material.

**Figure 4:**
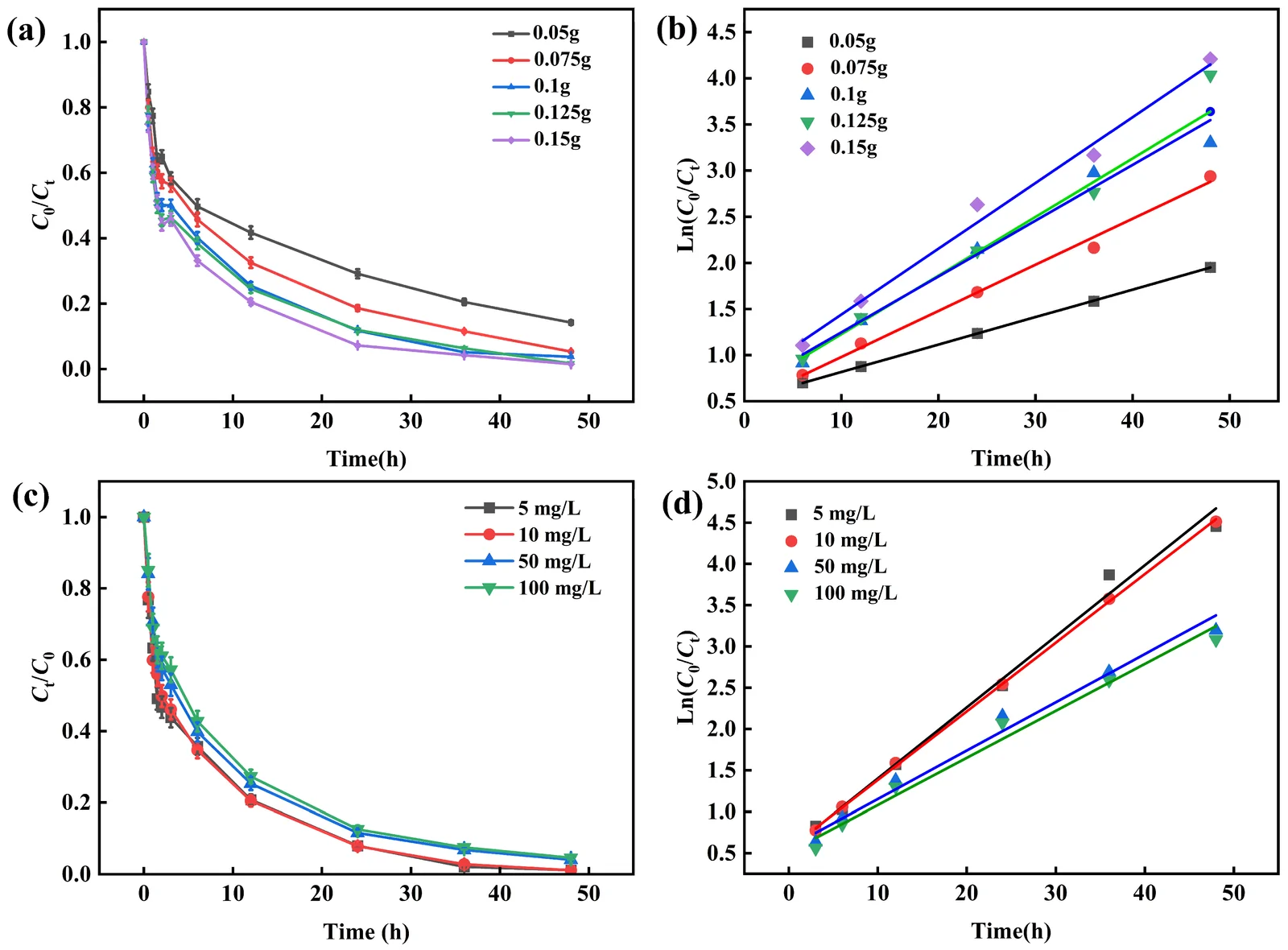
Effects of material dosage and initial perchlorate concentration on aqueous ClO_4_^−^ removal by nZVI@Mg(OH)_2_: (**a**) ClO_4_^−^ removal profiles and (**b**) corresponding pseudo−first−order kinetic fits at different material dosages (initial ClO_4_^−^ = 10 mg·L^−1^); (**c**) ClO_4_^−^ removal profiles and (**d**) corresponding pseudo−first−order kinetic fits at various initial ClO_4_^−^ concentrations (material dosage = 4.35 g·L^−1^). Experimental conditions: initial pH = 4.0 and T = 313 K. Experimental data in (**a**,**c**) are presented as mean ± SD (*n* = 3); some error bars are smaller than the data symbols. The symbols in (**b**,**d**) represent experimentally derived ln(*C*_0_/*C*_t_) values, while the solid lines indicate linear fits to the pseudo−first−order kinetic model.

The effect of material dosage was subsequently evaluated over the range of 0.05–0.15 g, corresponding to 2.17–6.51 g·L^−1^ (Figure 4a,b). Increasing the nZVI@Mg(OH)_2_ dosage progressively increased the apparent pseudo−first−order rate constant, *k_obs_*, from 0.031 to 0.075 h^−1^. Meanwhile, the corresponding half−life decreased from 22.36 to 9.24 h (Table S3). The positive dosage dependence can be attributed primarily to the increased inventory of accessible reactive interfaces and electron−donating Fe^0^ available within the reaction system [21,36,37]. However, the increase in *k_obs_* became less pronounced above 0.10 g. Such diminishing enhancement at high particle loadings is consistent with the tendency of nZVI suspensions to undergo secondary aggregation, which can decrease the fraction of accessible surface sites and increase local mass−transfer resistance [34,38,39]. Thus, increasing the material dosage enhances perchlorate reduction, but the kinetic benefit becomes progressively smaller at higher solid loadings.

The initial ClO_4_^−^ concentration also strongly influenced the apparent kinetics (Figure 4c,d). Across the concentration range of 5–100 mg·L^−1^, the aqueous ClO_4_^−^ disappearance profiles were well described by the pseudo−first−order model. The apparent rate constant, *k_obs_*, decreased from 0.084 h^−1^ at 5 mg·L^−1^ to 0.058 h^−1^ at 100 mg·L^−1^ (Table S4). The corresponding half−life increased from 8.25 to 11.95 h. At a fixed material dosage, a higher ClO_4_^−^ concentration increases competition for the available reaction sites and reducing equivalents [4,5,40]. This competition lowers the apparent first−order rate constant. Nevertheless, more than 95% of ClO_4_^−^ was removed within 48 h even at an initial concentration of 100 mg·L^−1^. This sustained removal over a 20−fold increase in substrate concentration indicates that the reconstructed core–shell interface remained accessible under the tested loading range. Together with the mesoporous architecture identified in Section 3.1, these results suggest that the Mg(OH)_2_−modified structure can maintain effective substrate transport to Fe−containing reaction interfaces even as the initial perchlorate concentration increases.

#### 3.2.2. Effects of Solution pH and Coexisting Anions

**Figure 5:**
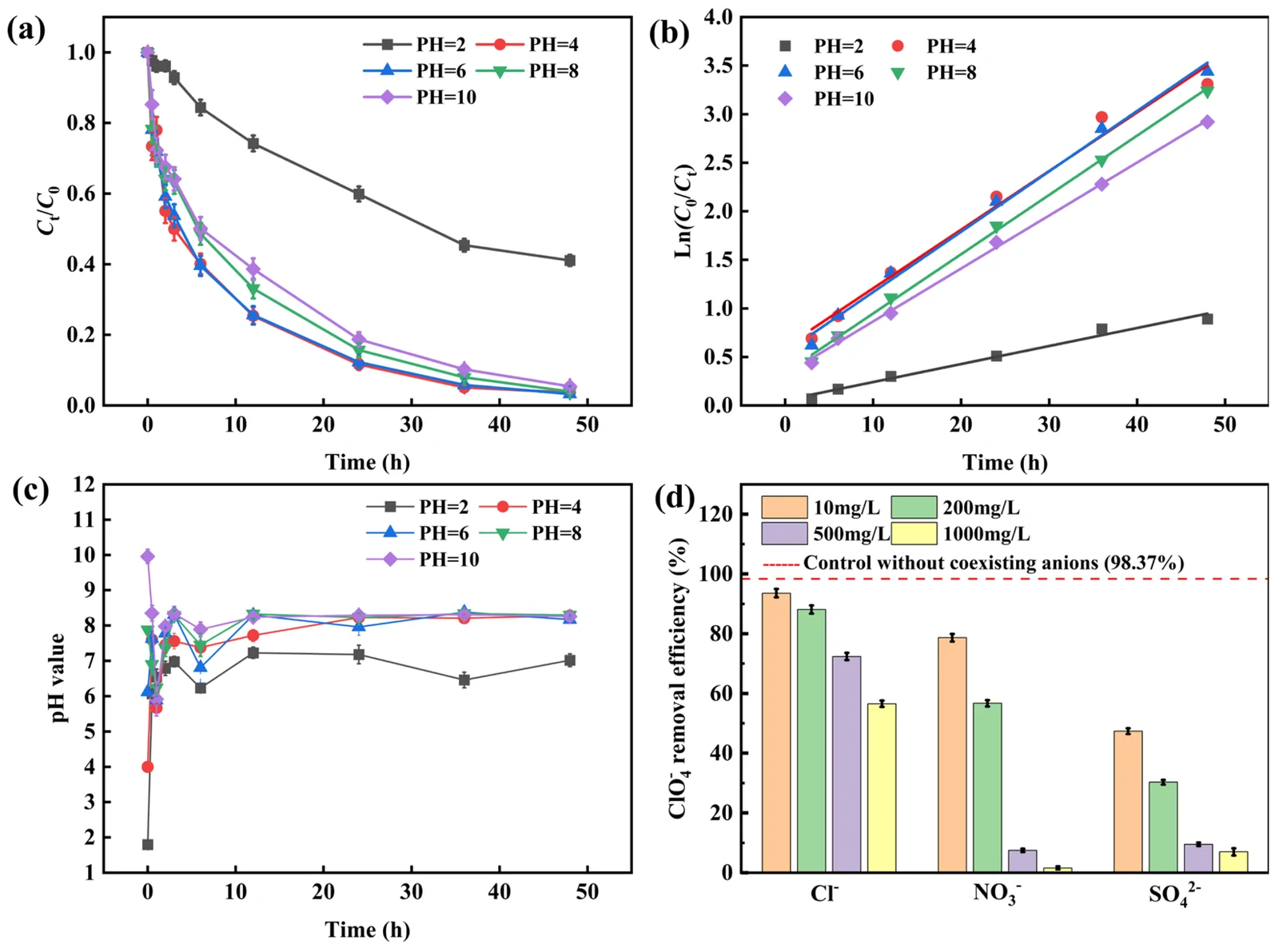
Effects of solution chemistry on ClO_4_^−^ removal by nZVI@Mg(OH)_2_: (**a**) ClO_4_^−^ removal profiles and (**b**) corresponding pseudo−first−order kinetic fits at different initial pH values; (**c**) time−resolved evolution of solution pH during the corresponding experiments; and (**d**) ClO_4_^−^ removal efficiencies after 48 h in the presence of Cl^−^, NO_3_^−^, and SO_4_^2−^ at different concentrations. The measured initial pH values in (**a**–**c**) were 1.80, 4.00, 6.12, 7.88, and 9.96, corresponding to nominal target values of 2, 4, 6, 8, and 10, respectively. The symbols in subfigure (**b**) represent experimentally derived ln(*C*_0_/*C*_t_) values, while the solid lines indicate linear fits to the pseudo−first−order kinetic model. The horizontal dashed line in (**d**) represents the 48 h ClO_4_^−^ removal efficiency of the control without added coexisting anions (98.37%) under otherwise identical conditions. Experimental conditions: material dosage = 4.35 g·L^−1^, initial ClO_4_^−^ concentration = 10 mg·L^−1^, and T = 313 K; for panel (**d**), initial pH = 4.0 and reaction time = 48 h.

Initial pH significantly affected the apparent ClO_4_^−^ removal kinetics (Figure 5a,b and Table S5). The measured kobs values were 0.019, 0.067, 0.068, 0.063, and 0.057 h−1 at measured initial pH values of 1.80, 4.00, 6.12, 7.88, and 9.96, respectively. The highest and most similar reaction rates were obtained over the moderately acidic−to−near−neutral range, whereas stronger inhibition occurred under the initially highly acidic condition. Time−resolved pH measurements provide additional insight into this behavior (Figure 5c). Rather than remaining at their initial values, all systems underwent rapid pH adjustment during the early reaction period. In the initially strongly acidic system, the pH increased from 1.80 to 6.07 within 0.5 h and subsequently remained mainly within approximately 6.2–7.2, reaching 7.02 after 48 h. The systems initially adjusted to pH 4.00–9.96 also showed rapid early variations and progressively converged toward a relatively narrow near−neutral to mildly alkaline range. Their final pH values were 8.28, 8.17, 8.30, and 8.26, respectively. Thus, the initial pH primarily determines the early interfacial chemical environment, whereas the later reaction proceeds within a substantially narrower pH range. The rapid increase in pH under the initially strongly acidic condition is consistent with proton−consuming processes associated with Fe^0^ corrosion and acid−driven dissolution of the Mg(OH)_2_−containing outer phase [28,34,37]. Strong acidity can also promote proton reduction and hydrogen evolution, diverting reducing equivalents from productive ClO_4_^−^ transformation [22,27,34,41].

Although the bulk solution rapidly approached near−neutral conditions, exposure to the strongly acidic initial environment may alter the Mg−containing outer region and the early Fe^0^ corrosion/passivation behavior, which is consistent with the substantially lower kobs observed for this condition. At the initially alkaline condition, the solution pH also rapidly shifted toward the same mildly alkaline range observed for the other systems. Therefore, the somewhat lower kobs at an initial pH of 9.96 is more appropriately associated with the early interfacial state established under alkaline conditions rather than with sustained high bulk pH throughout the reaction. Deprotonation of hydroxylated surface groups and the early formation of Fe (oxyhydr)oxide phases may reduce ClO_4_^−^ accessibility to reactive Fe−containing regions [37]. Overall, the pH profiles indicate that the observed pH dependence arises from the coupled evolution of Fe^0^ corrosion, Mg(OH)_2_ stability, surface protonation/deprotonation, and interfacial accessibility, with their relative importance changing as the solution pH self−adjusts during reaction.

Coexisting anions produced pronounced and concentration−dependent effects on ClO_4_^−^ removal (Figure 5d and Table S6). For direct comparison, the 48 h removal efficiency obtained in the absence of added coexisting anions under the benchmark conditions (98.37%) is shown as a horizontal dashed reference line in Figure 5d. Cl^−^ exerted the weakest interference over the investigated concentration range, whereas both SO_4_^2−^ and NO_3_^−^ substantially suppressed ClO_4_^−^ removal as their concentrations increased. The corresponding removal efficiencies at each anion concentration are summarized in Table S6. At the highest tested concentration of 1000 mg·L^−1^, SO_4_^2−^ reduced the ClO_4_^−^ removal efficiency to 7.00%, demonstrating severe inhibition under sulfate−rich conditions. Sulfate can interact strongly with hydroxylated Fe− and Mg−containing surfaces through surface complexation [42], thereby competing for accessible interfacial sites. The accompanying increase in ionic strength may further modify the electrical double layer and reduce the accessibility of ClO_4_^−^ to the reactive interface. These effects account for the pronounced deterioration in perchlorate removal observed at elevated sulfate concentrations. NO_3_^−^ also caused substantial inhibition, particularly at high concentrations. Despite its favorable thermodynamic reduction potential, ClO_4_^−^ remains kinetically resistant to reduction because of the high activation barrier associated with Cl−O bond cleavage [43,44,45].

Consequently, a kinetically more accessible electron acceptor such as NO_3_^−^ can competitively consume electrons released from Fe^0^, thereby decreasing the reducing equivalents available for ClO_4_^−^ transformation. Accordingly, the strong inhibition produced by NO_3_^−^ is most reasonably attributed to competition for reactive interfaces and reducing equivalents. Overall, the coexisting−anion experiments show that the performance of nZVI@Mg(OH)_2_ depends on both the type and concentration of background ions. High concentrations of sulfate and nitrate caused the strongest inhibition and therefore represent important constraints on perchlorate reduction.

#### 3.2.3. Temperature Dependence and Apparent Activation Energy

**Figure 6:**
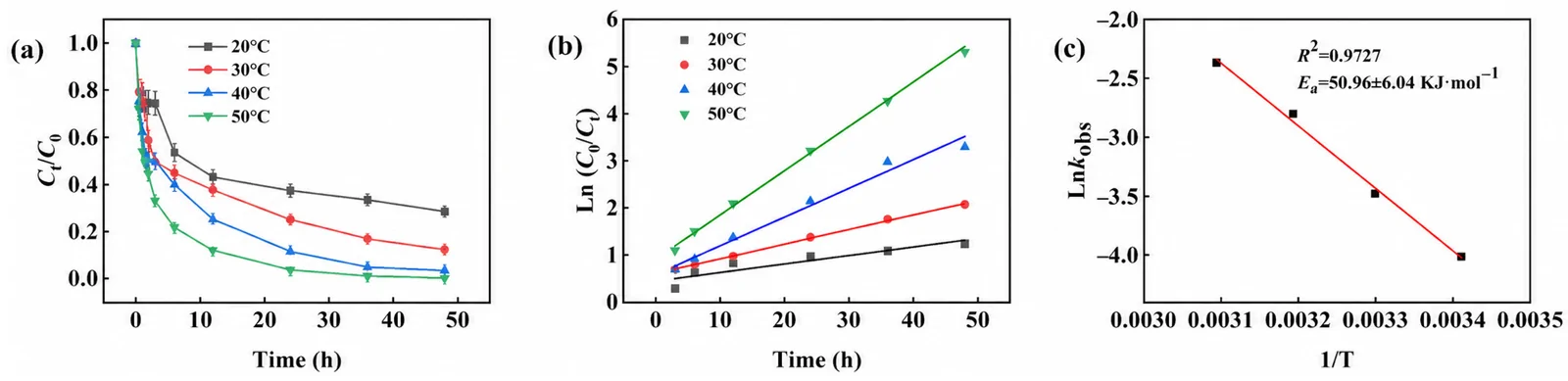
Effect of reaction temperature on ClO_4_^−^ removal by nZVI@Mg(OH)_2_: (**a**) aqueous ClO_4_^−^ removal profiles, (**b**) corresponding pseudo−first−order kinetic fits, and (**c**) linearized Arrhenius plot of ln*k*_obs_ as a function of 1/T. In (**a**), the colored symbols represent experimental data, the solid lines connect the data points to illustrate the concentration trends, and the error bars indicate the standard deviations of triplicate measurements. In (**b**), the symbols represent experimentally derived ln(*C*_0_/*C*_t_*)* values, while the solid lines indicate linear fits to the pseudo−first−order kinetic model. In (**c**), the black squares represent the calculated ln k_obs values, and the red solid line represents the linear regression based on the Arrhenius equation. Different colors in (**a**,**b**) correspond to different reaction temperatures, as indicated in the legends. Experimental conditions: material dosage = 4.35 g·L^−1^, initial ClO_4_^−^ = 10 mg·L^−1^, and initial pH = 4.0.

Temperature exerted a pronounced effect on the reduction kinetics of ClO_4_^−^ by nZVI@Mg(OH)_2_ (Figure 6a,b). As the reaction temperature increased from 20 to 50 °C, the apparent rate constant, *k_obs_*, increased from 0.021 to 0.153 h^−1^. This change corresponds to an approximately 7.3−fold increase in reaction rate. At the same time, the half−life decreased from 33.01 to 4.53 h (Table S7). The strong temperature dependence indicates that perchlorate reduction in this system involves a substantial kinetic barrier, consistent with the well−established kinetic inertness of the highly symmetric ClO_4_^−^ oxyanion.

The temperature dependence was quantified using the Arrhenius relationship between ln*k*_obs_ and 1/T (Figure 6c). The linear regression gave an *R*^2^ value of 0.9727. The calculated apparent activation energy (*E*_a_) was 50.96 ± 6.04 kJ·mol^−1^. The magnitude of this activation energy indicates that the overall reaction is not governed solely by bulk−phase diffusion and that an activated interfacial chemical process makes a substantial contribution to rate control. This interpretation agrees with the strong temperature dependence observed experimentally. It also provides kinetic evidence that ClO_4_^−^ reduction involves reaction steps occurring at or near the solid–liquid interface.

The apparent *E*_a_ obtained in this study (50.96 ± 6.04 kJ·mol^−1^) was lower than the value of 79.02 ± 7.75 kJ·mol^−1^ previously reported for perchlorate reduction by bare nZVI [4]. Because the two values were obtained under different experimental conditions, this comparison should not be interpreted as a direct quantitative measure of the Mg(OH)_2_ coating effect. Nevertheless, the lower apparent activation barrier observed for nZVI@Mg(OH)_2_ is consistent with a more accessible interfacial reaction environment. The reconstructed porous interface appears to preserve substrate accessibility while providing an interfacial environment favorable for the subsequent electron−transfer and reduction steps. The origin of this enhanced interfacial reactivity is further examined through phase−evolution and electrochemical analyses in Section 3.3.

### 3.3. Interfacial Phase Evolution and Electron−Transfer Behavior

#### 3.3.1. Phase Evolution During Perchlorate Treatment

**Figure 7:**
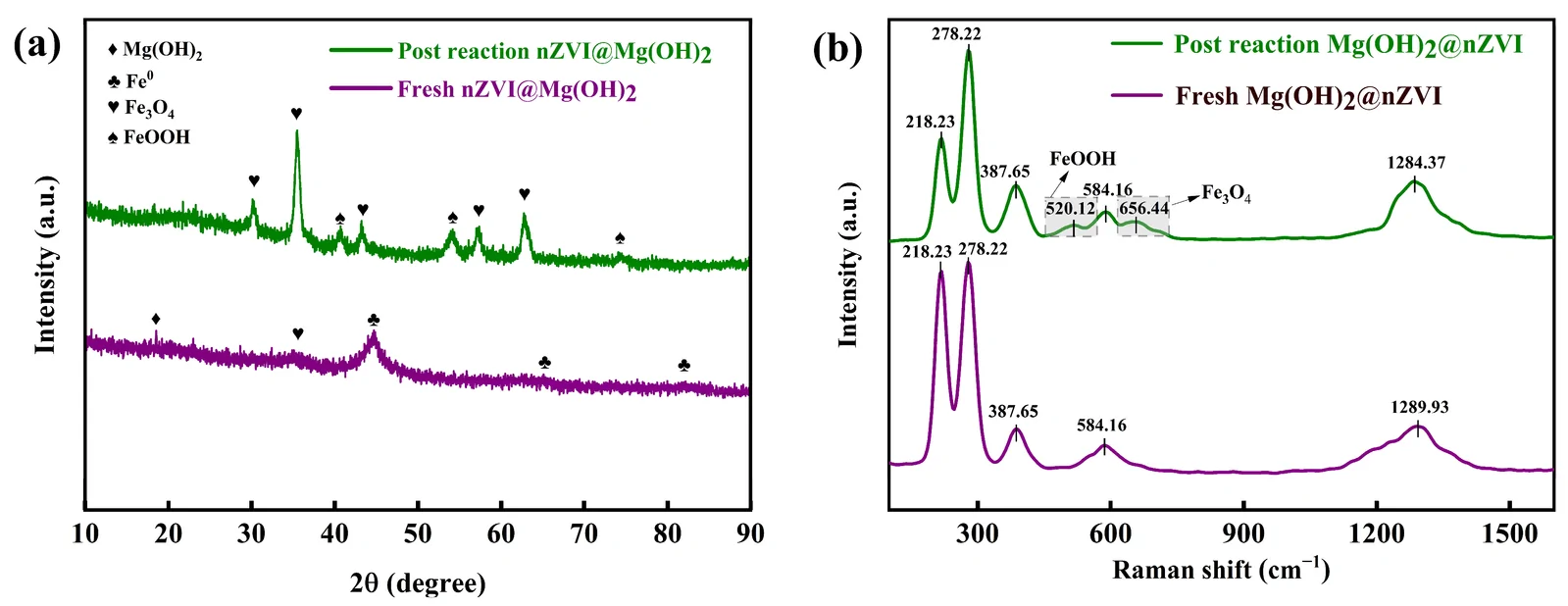
Phase evolution of nZVI@Mg(OH)_2_ before and after perchlorate treatment: (**a**) XRD patterns and (**b**) Raman spectra. In (**a**), characteristic reflections assigned to Mg(OH)_2_, α−Fe^0^, Fe_3_O_4_, and FeOOH are indicated by ♦, ♣, ♥ and ♠, respectively. In (**b**), the 100–1600 cm^−1^ region of the Raman spectra is shown for clarity; the complete spectra were acquired over 100–4000 cm^−1^.

The changes in Fe−containing phases during perchlorate treatment were examined by XRD, HRTEM, Raman spectroscopy, and FTIR. As shown in Figure 7a, fresh nZVI@Mg(OH)_2_ exhibited the dominant α−Fe^0^(110) reflection at approximately 2θ = 44.7°, together with weak low−intensity features near 18.5° and 38.0° that coincide with characteristic reflections of the pristine Mg(OH)_2_ reference. After reaction with ClO_4_^−^, the α−Fe^0^ reflection decreased markedly, while new reflections appeared at 2θ = 30.21°, 35.56°, 43.24°, 57.20°, and 62.81°. These reflections were assigned to the (220), (311), (400), (511), and (440) planes of spinel Fe_3_O_4_, respectively (PDF#19−0629). Two additional weak reflections around 40.7° and 74.3° were also discernible in the post−reaction pattern, and these two reflections were assigned to the (101), (102), and (202) planes of δ−Fe−OOH, respectively (JCPDS 13−0087). The pronounced decrease in the Fe^0^ signal together with the emergence of the Fe_3_O_4_ reflections supports substantial consumption of crystalline Fe^0^ and the development of Fe_3_O_4_ as the dominant crystalline Fe−containing reaction product. Post−reaction HRTEM further confirmed the formation of Fe_3_O_4_ at the lattice scale (Figure S3). The reacted nZVI@Mg(OH)_2_ showed lattice spacings of about 0.220, 0.256, and 0.295 nm. These values were assigned to the (400), (311), and (220) planes of Fe_3_O_4_, respectively. The observed lattice fringes confirm the formation of crystalline Fe_3_O_4_ during perchlorate treatment. They also show that the Fe−containing interface was strongly reconstructed as Fe^0^ was consumed. Although the spectra were acquired over 100–4000 cm^−1^, Figure 7b displays the 100–1600 cm^−1^ region containing the diagnostically relevant bands discussed here. For fresh nZVI@Mg(OH)_2_, bands at 218.23, 278.22, and 387.65 cm^−1^ were assigned to a thin hematite−like surface oxidation layer, while the feature at 584.16 cm^−1^ was associated with Fe−O vibrations in mixed−valence or poorly crystalline iron oxides. After perchlorate reaction, additional features at 520.12 and 656.44 cm^−1^ became evident. The band near 656.44 cm^−1^ is consistent with the symmetric Fe−O vibration of magnetite [46], whereas the feature near 520.12 cm^−1^ indicates a contribution from an additional hydroxylated and poorly crystalline Fe−containing phase. This Raman assignment is consistent with the post−reaction XRD and HRTEM observations.

The FTIR spectra further showed the formation of oxygen−containing Fe phases during the reaction (Figure S4). Before the reaction, broad bands at 3444.21 and 1635.12 cm^−1^ were assigned to surface hydroxyl groups and adsorbed water, respectively. After the reaction, these bands shifted slightly to 3439.73 and 1631.08 cm^−1^. A new band also appeared at 577.08 cm^−1^. This Fe−O vibration shows the formation of iron oxide phases during Fe^0^ oxidation. It also supports the interfacial reconstruction identified by XRD, HRTEM, and Raman spectroscopy.

Taken together, the post−reaction XRD, HRTEM, Raman, and FTIR results support pronounced reconstruction of the Fe−containing interface. Progressive consumption of Fe^0^ is accompanied by the formation of a crystalline Fe_3_O_4_−dominated reaction layer together with a smaller contribution from poorly crystalline hydroxylated Fe phases. This heterogeneous phase evolution establishes the structural basis for the altered interfacial electrochemical behavior discussed in Section 3.3.2.

#### 3.3.2. Interfacial Electrochemical Behavior

**Figure 8:**
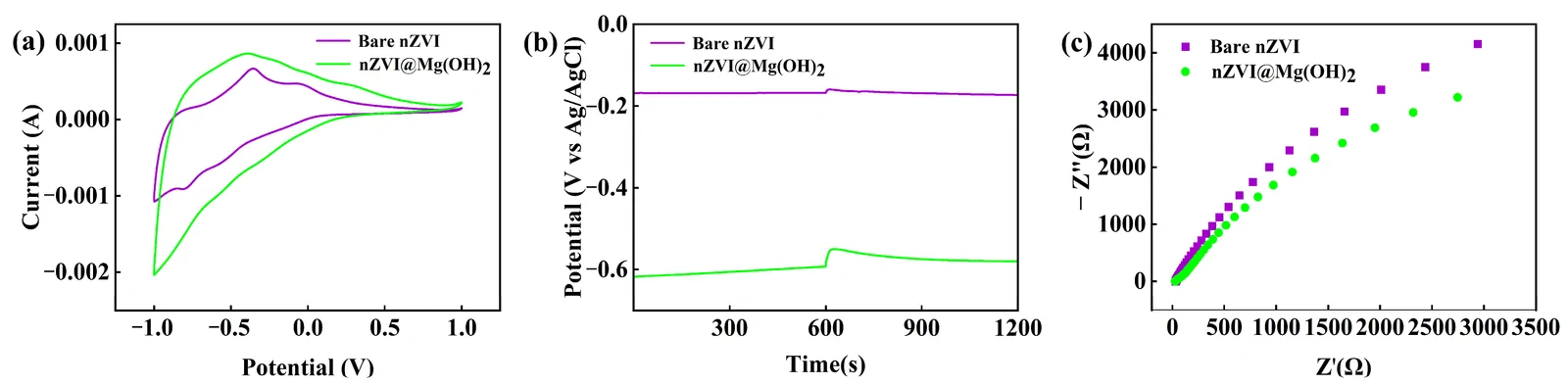
Electrochemical characterization of bare nZVI and nZVI@Mg(OH)_2_: (**a**) cyclic voltammetry curves; (**b**) OCP−time profiles, with ClO_4_^−^ introduced at approximately 600 s; and (**c**) EIS Nyquist plots. Detailed fitted parameters are provided in Table S8.

Electrochemical measurements were subsequently performed to determine whether the structural and phase evolution of the core–shell interface was accompanied by changes in interfacial electron−transfer behavior. As shown by the CV profiles in Figure 8a, nZVI@Mg(OH)_2_ showed a markedly different current response from bare nZVI. It also exhibited a broader redox response. These changes demonstrate that the Mg(OH)_2_ shell substantially modified the electrochemical behavior of the Fe−containing interface. Importantly, the coating did not simply suppress the electrochemical accessibility of the Fe core, as would be expected for a compact insulating layer.

This distinction was further supported by the in situ OCP measurements (Figure 8b). The steady−state OCP shifted from approximately −0.168 V for bare nZVI to −0.617 V for nZVI@Mg(OH)_2_ versus Ag/AgCl, indicating that the modified material maintained a substantially more reducing interfacial electrochemical state. Importantly, ClO_4_^−^ was introduced directly into the electrochemical cell after approximately 600 s of baseline equilibration. The nZVI@Mg(OH)_2_ electrode exhibited an immediate transient potential perturbation followed by rapid restabilization. This response demonstrates rapid electrochemical coupling between ClO_4_^−^ addition and the accessible solid–liquid interface [35,37,47]. Although the OCP response does not by itself resolve the elementary electron−transfer step, its occurrence directly upon ClO_4_^−^ addition provides complementary evidence that the reconstructed interface responds electrochemically to the target oxyanion.

The EIS results further resolved the effect of Mg(OH)_2_ modification on the interfacial electrochemical response (Figure 8c). Equivalent−circuit analysis showed comparable solution resistances for bare nZVI and nZVI@Mg(OH)_2_ (*R*_s_ = 27.48 and 26.03 Ω, respectively), indicating that the difference between the two spectra was not primarily caused by bulk electrolyte resistance. Bare nZVI was adequately described by a single interfacial relaxation element, *R*_s_ − (*R*_1_//*CPE*_1_), with *R*_1_ = 15.21 kΩ. In contrast, the modified electrode required two relaxation elements, *R*_s_ − (*R*_1_//*CPE*_1_) − (*R*_2_//*CPE*_2_), yielding *R*_1_ = 78.66 Ω and *R*_2_ = 10.70 kΩ. The corresponding total fitted polarization resistance decreased from 15.21 kΩ for bare nZVI to 10.78 kΩ for nZVI@Mg(OH)_2_, representing a decrease of approximately 29.1%. The fittings gave χ^2^ values of 0.00486 and 0.00197, respectively, indicating satisfactory agreement between the equivalent−circuit models and the measured impedance responses (Table S8).

The requirement for two relaxation processes in nZVI@Mg(OH)_2_ is consistent with the increased heterogeneity of the reconstructed Fe/Mg−containing interface, whereas the lower overall polarization resistance indicates greater electrochemical accessibility of the modified interface. EIS has previously been used to resolve interfacial reaction and electron−transfer behavior in ZVI systems [35], and the present fitted results similarly show that Mg(OH)_2_ modification does not produce a uniformly blocking insulating layer. Rather, when considered together with the mesoporous structure and Fe−phase reconstruction described above, the lower fitted interfacial resistance is consistent with more accessible interfacial electron−transfer pathways [27,35,48]. The electrochemical measurements were conducted in 0.1 M Na_2_SO_4_ to provide the same supporting−electrolyte environment for both electrodes. Sulfate can interact with hydroxylated Fe−containing surfaces through surface complexation, and the batch experiments in Section 3.2.2 indeed showed strong inhibition of ClO_4_^−^ removal at elevated SO_4_^2−^ concentrations. The absolute OCP and impedance values obtained in the electrochemical cell therefore reflect a sulfate−rich interfacial environment. Because bare nZVI and nZVI@Mg(OH)_2_ were measured under identical electrolyte composition, the EIS comparison is used here to evaluate their relative interfacial electrochemical behavior rather than to reproduce the absolute kinetics of the batch ClO_4_^−^ removal system. Taken together with the more negative OCP and the immediate potential response following ClO_4_^−^ addition, the fitted EIS results support a more electrochemically accessible Fe−containing interface after Mg(OH)_2_ modification.

#### 3.3.3. Surface Chemical−State Evolution

**Figure 9:**
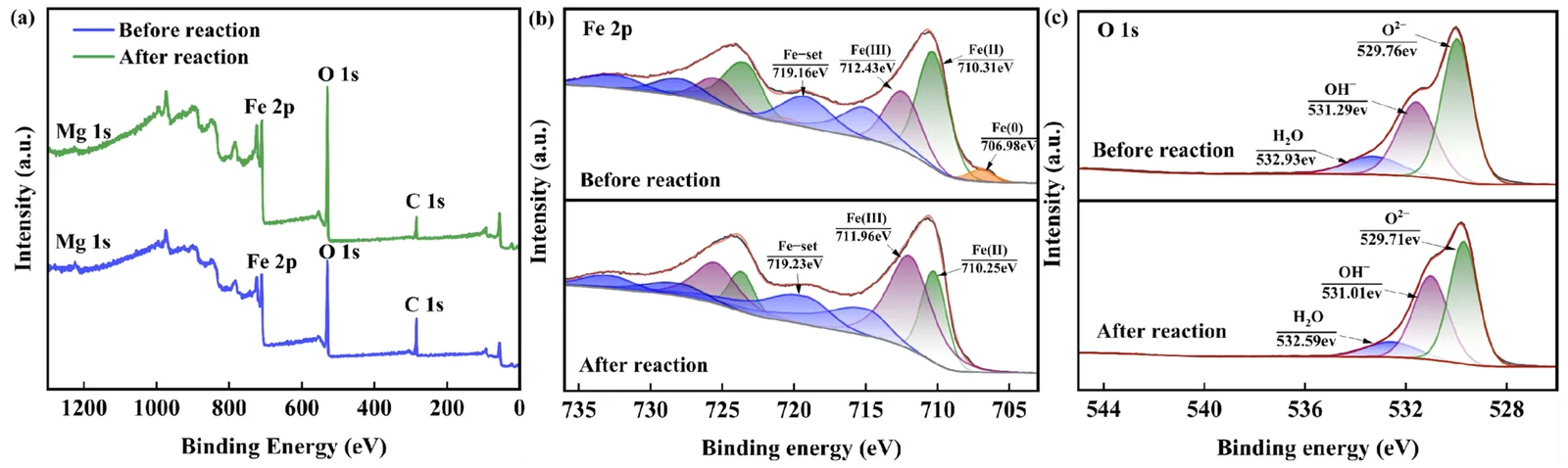
XPS characterization of nZVI@Mg(OH)_2_ before and after reaction with perchlorate: (**a**) survey spectra, (**b**) high−resolution Fe 2p spectra, and (**c**) high−resolution O 1s spectra. In (**a**), the blue and green lines represent the spectra before and after reaction, respectively. In (**b**,**c**), the colored shaded peaks represent the deconvoluted contributions of different chemical states, as identified by their corresponding labels and binding energies. The superimposed curves represent the experimental spectra and overall fitting results.

XPS was used to examine the Fe and O chemical states on the nZVI@Mg(OH)_2_ surface before and after reaction with perchlorate. The survey spectra (Figure 9a) showed that the overall elemental composition of the composite remained similar after reaction. However, the Fe and O chemical states changed clearly. These changes show that the Fe−containing surface layer was strongly reconstructed during perchlorate treatment.

The Fe 2p spectra provide clear evidence for progressive oxidation of the Fe−containing interface (Figure 9b and Table S9). Prior to reaction, the fitted Fe species consisted of Fe^0^ at 706.98 eV (4.95%), Fe(II) at 710.31 eV (60.13%), and Fe(III) at 712.43 eV (34.92%). After reaction, the surface−accessible Fe^0^ component was no longer detected, while the relative abundance of Fe(II) decreased to 34.10% at 710.25 eV and that of Fe(III) increased to 65.90% at 711.96 eV. This redistribution demonstrates pronounced oxidation of the Fe−containing surface and is consistent with progressive Fe^0^ oxidation accompanied by the formation of mixed Fe(II)/Fe(III) oxide phases and increasingly oxidized surface species [37,46,49,50]. Such Fe^0^ oxidation provides the reducing equivalents required for interfacial perchlorate transformation. The Fe(III)−enriched surface detected by XPS is compatible with the Fe_3_O_4_−dominated crystalline phase identified by XRD because XPS preferentially probes the outermost surface region, where hydroxylation and further oxidation are more pronounced.

Consistent with this interpretation, the O 1s spectra showed a clear increase in surface hydroxylation after reaction (Figure 9c and Table S10). The lattice−oxygen component decreased from 53.05% at 529.76 eV to 48.67% at 529.71 eV, whereas the hydroxylated−oxygen component increased from 34.83% at 531.29 eV to 40.20% at 531.01 eV. The contribution associated with adsorbed H_2_O changed only slightly, from 12.12% to 11.13%. The enrichment of hydroxylated oxygen is consistent with the development of Fe oxyhydroxide species at the outer solid–liquid interface and supports the minor FeOOH−like contribution inferred from Raman spectroscopy.

Taken together, the XPS results reveal progressive oxidation and hydroxylation of the outer Fe−containing surface during perchlorate treatment. In conjunction with the post−reaction XRD and Raman results, these observations support the formation of a heterogeneous reaction layer containing a Fe_3_O_4_−dominated crystalline component together with a more oxidized and hydroxylated outer surface. The complementary information obtained from XRD and XPS therefore provides a consistent description of Fe−phase transformation during reaction.

### 3.4. Interfacial Electron−Transfer Pathway and Proposed Reduction Mechanism

**Figure 10:**
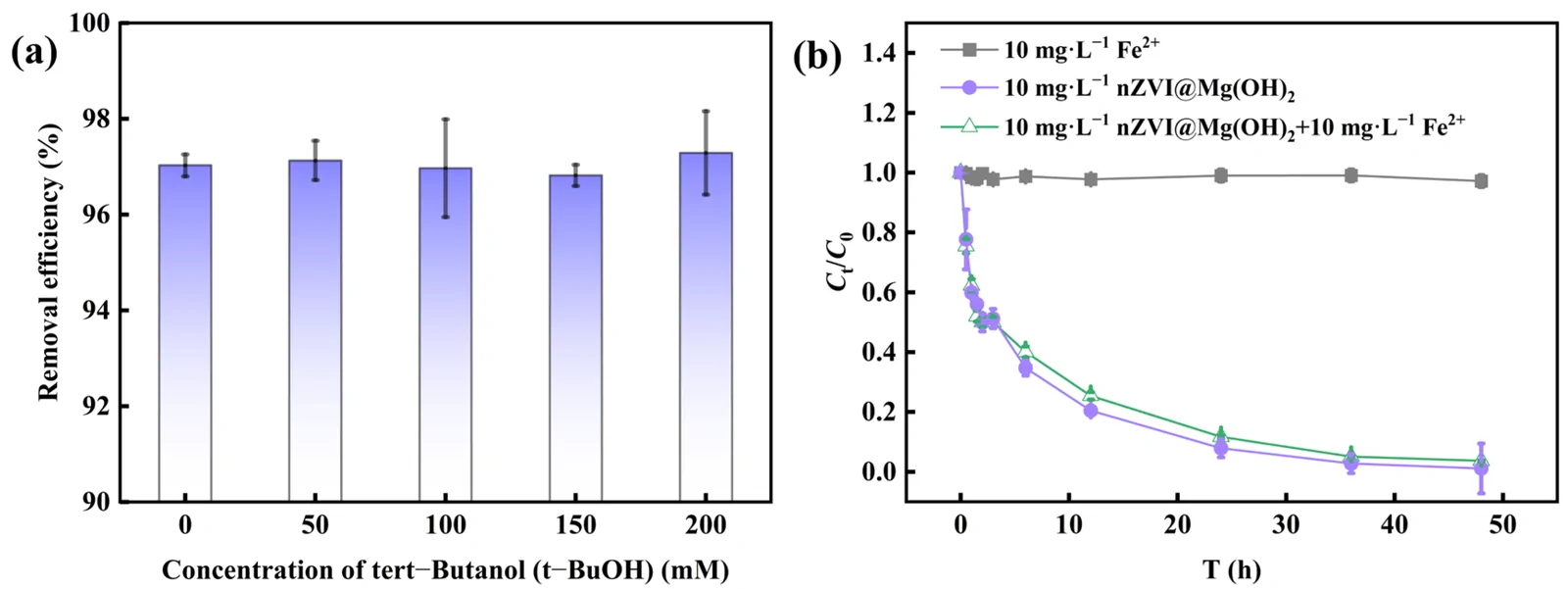
Mechanistic control experiments probing possible electron−transfer pathways during ClO_4_^−^ transformation by nZVI@Mg(OH)_2_: (**a**) effect of TBA concentration on ClO_4_^−^ removal; (**b**) ClO_4_^−^ removal in the presence of dissolved Fe^2+^ alone, nZVI@Mg(OH)_2_, and nZVI@Mg(OH)_2_/Fe^2+^ system. Experimental data are presented as mean ± SD (*n* = 3).

To evaluate the relative contributions of plausible electron−transfer pathways during ClO_4_^−^ transformation, scavenging and homogeneous−control experiments were interpreted together with the kinetic, electrochemical, and solid−phase characterization results. In Fe^0^−based systems, contaminant transformation may involve direct electron transfer at the solid–liquid interface as well as indirect pathways involving reactive hydrogen−containing species generated during Fe^0^ corrosion [27,29,51]. As shown in Figure 10a, increasing the TBA concentration from 0 to 200 mM produced no appreciable change in ClO_4_^−^ removal, with the final removal efficiency remaining approximately 97%. This weak response indicates that reactive species effectively quenched by TBA did not make a major contribution under the investigated conditions. However, because TBA can also influence interfacial chemistry and mass transport, the scavenging result alone cannot distinguish direct interfacial electron transfer from other heterogeneous reduction pathways. Accordingly, the TBA experiment is used here only to constrain the possible contribution of TBA−quenchable reactive species rather than as independent proof of direct electron transfer.

The possible contribution of dissolved Fe^2+^ was examined independently (Figure 10b). Addition of 10 mg·L^−1^ Fe^2+^ in the absence of nZVI@Mg(OH)_2_ resulted in negligible ClO_4_^−^ removal over 48 h, demonstrating that dissolved Fe^2+^ alone was ineffective toward perchlorate under the investigated conditions. Moreover, supplementing the nZVI@Mg(OH)_2_ system with the same concentration of Fe^2+^ produced no appreciable enhancement relative to the composite alone. These observations indicate that homogeneous reduction mediated by dissolved Fe^2+^ is not a major pathway under the investigated conditions. Together with the strong dependence of ClO_4_^−^ removal on the presence of the solid composite, the results point to a predominantly heterogeneous interfacial transformation process.

This interpretation is consistent with the kinetic, electrochemical, and solid−phase evidence presented above. The apparent activation energy of 50.96 ± 6.04 kJ·mol^−1^ shows that the overall transformation is not controlled solely by bulk−phase diffusion but involves an activated process at or near the solid–liquid interface. In parallel, nZVI@Mg(OH)_2_ exhibited a more negative OCP and lower interfacial impedance than bare nZVI. The immediate OCP perturbation following direct addition of ClO_4_^−^ further demonstrates electrochemical coupling between the target oxyanion and the reconstructed interface. XRD, Raman, and XPS additionally revealed progressive Fe^0^ consumption accompanied by the formation of a Fe_3_O_4_−dominated crystalline phase and a more oxidized and hydroxylated outer region. Taken together, these observations, along with the negligible contribution of dissolved Fe^2+^, are consistent with a predominantly heterogeneous, Fe^0^−driven reductive transformation at the solid–liquid interface. It should be emphasized, however, that the kinetic measurements directly quantify only the disappearance of aqueous ClO_4_^−^. A closed chlorine mass balance and quantitative distributions of Cl^−^, ClO_3_^−^, ClO_2_^−^, and other chlorine−containing products were not established. Therefore, the measured ClO_4_^−^ removal cannot be interpreted directly as a quantitatively resolved reduction yield, and contributions from adsorption or surface−associated retention cannot be completely excluded. Accordingly, the performance data are reported as ClO_4_^−^ removal, whereas the subsequent reduction pathway is presented as a mechanistic interpretation supported by the combined kinetic, electrochemical, and solid−phase evidence.

**Figure 11:**
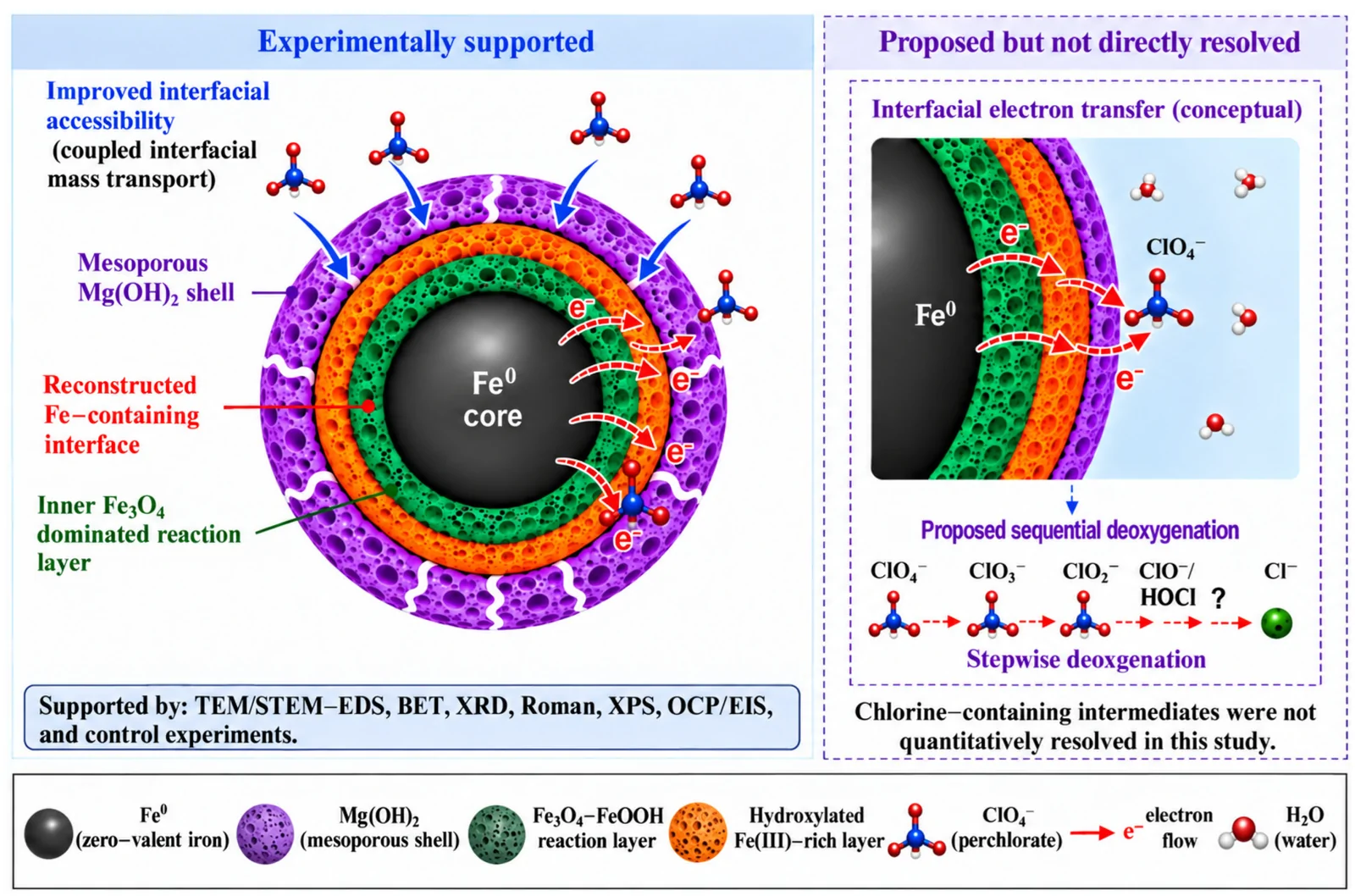
Conceptual mechanism proposed for ClO_4_^−^ transformation by nZVI@Mg(OH)_2_ through coupled interfacial mass transport and heterogeneous interfacial electron−transfer processes. The colors schematically distinguish the Fe^0^ core (black), Mg(OH)_2_−containing region (purple), Fe_3_O_4_−dominated reaction layer (green), and hydroxylated Fe(III)−rich region (orange), without implying uniformly continuous layers. Blue arrows indicate proposed ClO_4_^−^ transport toward Fe−containing interfaces, whereas red arrows indicate conceptual electron−transfer directions. Red dashed arrows in the right panel represent possible sequential chlorine−species transformations that were not independently resolved in this study.

On the basis of these observations, a conceptual mechanistic model for ClO_4_^−^ transformation by nZVI@Mg(OH)_2_ is proposed in Figure 11. First, deposition of the Mg(OH)_2_ shell suppresses severe magnetic aggregation of nZVI and generates an open mesoporous architecture with substantially increased accessible surface area. This structural reconstruction facilitates transport of ClO_4_^−^ from the aqueous phase toward Fe−containing reaction interfaces. Under acidic−to−near−neutral conditions, protonation of hydroxylated Mg(OH)_2_ surface groups may additionally increase the affinity of the interface toward anionic ClO_4_^−^, thereby favoring its local interfacial accumulation.

Second, the Mg(OH)_2_−modified architecture regulates the evolution of the Fe−containing interface during reaction. Rather than developing exclusively into a uniformly dense passivation layer, the reacted material contains a crystalline Fe_3_O_4_−dominated phase together with a more hydroxylated Fe(III)−enriched outer region. Combined with the lower charge−transfer resistance observed by EIS, this heterogeneous reaction layer remains compatible with continued interfacial electron transport during progressive oxidation of the Fe^0^ core. Thus, the role of the Mg(OH)_2_ shell is not simply physical protection of Fe^0^ but regulation of the accessibility and electrochemical properties of the evolving solid–liquid interface.

Finally, electrons released during Fe^0^ oxidation are proposed to participate in interfacial ClO_4_^−^ transformation. Successive electron−transfer and deoxygenation steps provide a chemically plausible pathway; however, the individual chlorine−containing intermediates and final chlorine−product distribution were not quantitatively resolved in the present study. Therefore, the sequence illustrated in Figure 11 should be regarded as a conceptual pathway rather than direct identification of the chlorine−product sequence.

## 4. Conclusions

In this study, a mesoporous nZVI@Mg(OH)_2_ core–shell−like composite was constructed to regulate substrate accessibility and interfacial electron−transfer behavior during perchlorate treatment. The composite prepared at a nominal Mg(OH)_2_/nZVI mass ratio of 10% effectively mitigated nZVI aggregation and reconstructed the particle architecture, increasing the BET specific surface area from 4.57 to 67.29 m^2^·g^−1^ and substantially enhancing textural mesoporosity. Under the optimized benchmark conditions, the composite achieved 98.37% aqueous−phase removal of 10 mg·L^−1^ ClO_4_^−^ within 48 h. This value represents aqueous−phase ClO_4_^−^ removal rather than a directly quantified reduction yield, because chlorine−containing transformation products were not quantified. The system maintained relatively stable reactivity over the moderately acidic−to−near−neutral initial pH range, while time−resolved measurements showed rapid pH self−adjustment toward near−neutral to mildly alkaline conditions during reaction. In contrast, high concentrations of SO_4_^2−^ and NO_3_^−^ substantially inhibited perchlorate removal. These results highlight the combined roles of solution chemistry, interfacial accessibility, and competition for reactive interfaces and reducing equivalents in controlling overall ClO_4_^−^ transformation.

Mechanistic analyses further showed that Mg(OH)_2_ modification altered the evolution of the Fe−containing interface rather than acting merely as a passive protective coating. During reaction, Fe^0^ was progressively consumed, and the interface evolved toward a Fe_3_O_4_−dominated crystalline reaction layer accompanied by a more oxidized and hydroxylated Fe(III)−rich outer region. This heterogeneous interfacial reconstruction was associated with a more reducing electrochemical state and a lower fitted total polarization resistance than bare nZVI, consistent with greater electrochemical accessibility of the reconstructed interface. The apparent activation energy of 50.96 ± 6.04 kJ·mol^−1^ further indicated a substantial contribution from activated interfacial chemical steps to the overall reaction kinetics.

Combined electrochemical, scavenging, and Fe^2+^ control experiments support a predominantly heterogeneous interfacial transformation process under the investigated conditions. The absence of a measurable TBA effect argues against a major contribution from TBA−quenchable reactive species, while the Fe^2+^ controls exclude a major homogeneous Fe^2+^−mediated pathway. Together with the immediate OCP response upon ClO_4_^−^ addition and the lower interfacial impedance of nZVI@Mg(OH)_2_, these observations are consistent with a major contribution from interfacial electron transfer, with direct electron transfer representing a plausible electron−delivery route.

Accordingly, the enhanced reactivity of nZVI@Mg(OH)_2_ is attributed to the coupling of improved substrate accessibility with an electronically accessible, low−resistance Fe−containing interface. These findings provide mechanistic insight into how coordinated regulation of mass transport and interfacial electron−transfer behavior can improve the reactivity of Fe−based materials toward kinetically persistent oxyanions. Given the progressive Fe^0^ consumption observed during reaction, future work should further quantify dissolved Fe and Mg release and evaluate the feasibility of material reuse or regeneration to better assess long−term stability and potential secondary contamination.

## Data Availability

The data presented in this study are available on request from the corresponding author.

## Supplementary Materials

The following supporting information can be downloaded at: https://www.mdpi.com/article/10.3390/nano16211364/s1, Text S1–S3, Figures S1–S4 and Tables S1–S10 are included.
