# Supplementary material for: Interface Reconstruction of nZVI with a Mesoporous Mg(OH)2 Shell: Coupled Mass Transport and Interfacial Electron Transfer During Perchlorate Transformation
Source: Nanomaterials (Basel). 2026 Oct 26;16(21):1364. doi: 10.3390/nano16211364 (PMC13764807; doi:10.3390/nano16211364)
Supplement: Supplementary file 1 [file nanomaterials-16-01364-s001.zip › nanomaterials-4595228-supplementary.pdf]

Supplementary Material For
Interface Reconstruction of nZVI with a Mesoporous $Mg(OH)_2$ Shell: Coupled Mass Transport and Interfacial Electron Transfer during Perchlorate Transformation

Keke Xu [1], Yongkang Sun [2, 3], Lichao Tan [1], Mingzhu Zhang [1], Wenming Pei [1], Ziyan Du [1], Jiqiang Jia [1], Xiaoyan Zhang [1], Jingjing Yun [1], Feng Ge [1, *], Lei Wang [1, *], Ningyuan Zhu [2, *]

[1] *Key Laboratory of Pesticide Environmental Assessment and Pollution Control, Nanjing Institute of Environmental Science, Ministry of Ecology and Environment of the People's Republic of China, Nanjing 210042, China; xukeke@nie.org (K.X.)*

[2] College of Environment, *Hohai University, Nanjing 210098, China; xyhbj@126.com*

[3] *Xuyi Ecological Environment Comprehensive Administrative Law Enforcement Bureau, Huai'an, Huaian 211700, China*

*Correspondence: gefeng@nies.org (F.G.); wlofrcc@126.com (L.W.); zhuny0106@hhu.edu.cn (N.Z.)

The following are included as supporting information for this paper:

number of pages: 16

number of texts: 5

number of figures: 3

number of tables: 5

Texts

Text S1. Chemicals and reagents.

Text S2. Preparation of solutions.

Text S3. Material characterization and electrochemical methods.

Figures

Fig. S1. Morphology and elemental distribution of nZVI@Mg(OH)$_2$.

Fig. S2. Effect of Mg(OH)$_2$ loading on perchlorate removal by bare nZVI and nZVI@Mg(OH)$_2$ composites.

Fig. S3. TEM and HRTEM images of nZVI@Mg(OH)$_2$ after perchlorate reaction.

Fig. S4. FTIR spectra of nZVI@Mg(OH)$_2$ before and after perchlorate reaction.

Tables

Table S1. Specific surface area and pore−structure parameters of bare nZVI and nZVI@Mg(OH)$_2$.

Table S2. BET specific surface area and 48 h ClO_4^- removal efficiency between B,N−doped porous−carbon−supported nZVI and nZVI@Mg(OH)$_2$.

Table S3. Pseudo−first−order kinetic parameters for ClO_4^- removal by nZVI@Mg(OH)$_2$ at different material dosages.

Table S4. Pseudo−first−order kinetic parameters for ClO_4^- removal by nZVI@Mg(OH)$_2$ at different initial ClO_4^- concentrations.

Table S5. Pseudo−first−order kinetic parameters for ClO_4^- removal by nZVI@Mg(OH)$_2$ at different initial pH values.

Table S6. Effects of coexisting anions on perchlorate removal by nZVI@Mg(OH)$_2$.

Table S7. Pseudo−first−order kinetic parameters for ClO_4^- removal by nZVI@Mg(OH)$_2$ at different reaction temperatures.

Table S8. Equivalent−circuit fitting parameters for EIS spectra of bare nZVI and nZVI@Mg(OH)$_2$.

Table S9. Fe 2p XPS fitting results for nZVI@$Mg(OH)_2$ before and after perchlorate reaction.

Table S10. O 1s XPS fitting results for nZVI@$Mg(OH)_2$ before and after perchlorate reaction.

Text S1. Chemicals and reagents

All chemicals were of analytical grade and were used without further purification. Iron(III) chloride hexahydrate ($FeCl_3·6H_2O$, 99%), potassium perchlorate ($KClO_4$, 99%) and ferrous sulfate monohydrate ($FeSO_4·H_2O$, 98%) were purchased from Shanghai Macklin Biochemical Technology Co., Ltd. Magnesium hydroxide ($Mg(OH)_2$, 95%) and tert-butyl alcohol (TBA, $C_4H_{10}O$, 99.5%) were obtained from Shanghai Aladdin Biochemical Technology Co., Ltd. Sodium borohydride ($NaBH_4$, 98%), sodium chloride (NaCl, ≥99.5%), sodium sulfate (Na_2SO_4, 99%), and sodium nitrate ($NaNO_3$, 99%) were supplied by Nanjing Chemical Reagent Co., Ltd. Absolute ethanol (C_2H_5OH, ≥99.7%), hydrochloric acid (HCl, 36−38%), isopropanol (C_3H_8O, ≥99.7%), and sodium hydroxide (NaOH, ≥96%) were purchased from Sinopharm Chemical Reagent Co., Ltd. Nafion solution (5 wt%) and isopropanol were used for preparation of the electrochemical working electrodes. Ultrapure deionized water (18.2 MΩ·cm) was used throughout the experiments. Deoxygenated water was prepared by purging deionized water with high−purity N_2 before use.

Text S2. Preparation of solutions

- **Fe(III) precursor solution:** $FeCl_3·6H_2O$ (4.889 g) was dissolved in 20 mL of deoxygenated deionized water to prepare the Fe(III) precursor solution used for nZVI synthesis.

- **$Mg(OH)_2$ dispersion:** $Mg(OH)_2$ was dispersed in 80 mL of absolute ethanol and ultrasonicated for 10 min before mixing with the Fe(III) precursor solution. The amount of $Mg(OH)_2$ was adjusted to obtain nominal $Mg(OH)_2$/nZVI feed mass ratios of 1%, 10%, and 20%, defined relative to the theoretical Fe^0 mass calculated from the amount of $FeCl_3·6H_2O$ precursor. For the nominal 10% formulation, 4.889 g

$FeCl_3 \cdot 6H_2O$ (18.09 mmol Fe) corresponds to approximately 1.010 g theoretical Fe^0, and 0.105 g $Mg(OH)_2$ (1.80 mmol) therefore gives an $Mg(OH)_2$/nZVI feed mass ratio of approximately 10.4%.

- **$NaBH_4$ reducing solution:** $NaBH_4$ (2.735 g) was dissolved in 100 mL of deoxygenated deionized water. The reducing solution was freshly prepared immediately before use.
- **Perchlorate stock solution:** A perchlorate stock solution with a nominal concentration of 1000 $mg \cdot L^{-1}$, expressed as ClO_4^-, was prepared from $KClO_4$ using deoxygenated deionized water. Working solutions with the required ClO_4^- concentrations were obtained by appropriate dilution of the stock solution.
- **Electrochemical supporting electrolyte:** A 0.1 $mol \cdot L^{-1}$ Na_2SO_4 solution was prepared by dissolving 14.204 g of anhydrous Na_2SO_4 in deionized water and diluting to 1 L.
- **Fe(II) solution:** Fe^{2+} control experiments were conducted using ferrous sulfate monohydrate ($FeSO_4 \cdot H_2O$) as the Fe^{2+} source. Fe^{2+} was introduced at a final concentration of 10 $mg \cdot L^{-1}$ either in the absence of nZVI@$Mg(OH)_2$ or together with the composite under otherwise identical reaction conditions.
- **pH adjustment:** The initial solution pH was adjusted using 0.1 $mol \cdot L^{-1}$ HCl or 0.1 $mol \cdot L^{-1}$ NaOH.

Text S3. Material characterization and electrochemical methods

X−ray diffraction

X−ray diffraction (XRD) patterns were recorded using a Rigaku SmartLab SE diffractometer (Japan) with Cu K_α radiation (λ = 1.5406 Å). The instrument was operated at 40 kV and 30 mA. Diffraction patterns were collected over a 2θ range of

5−90° at a scanning rate of 5°·min^{-1}. It should be specifically noted that, to increase the counting time and improve the signal−to−noise ratio of weak diffraction features, the fresh nZVI@$Mg(OH)_2$ was collected at a scan rate of 1 °·min^{-1}. The pristine $Mg(OH)_2$ and bare nZVI patterns were used as reference patterns for phase comparison.

X−ray photoelectron spectroscopy

X−ray photoelectron spectroscopy (XPS) measurements were performed using a Thermo Scientific K−Alpha spectrometer (USA) equipped with a monochromatic Al K_α source (h_v = 1486.6 eV). Survey spectra and high−resolution Fe 2p, Mg 1s and O 1s spectra were analyzed in the present study. All binding energies were calibrated against the C 1s peak at 284.8 eV. Peak fitting was conducted using Thermo Avantage software.

Scanning electron microscopy and elemental mapping

Surface morphology and elemental distributions were examined using a ZEISS Sigma 300 field−emission scanning electron microscope equipped with energy−dispersive X−ray spectroscopy (SEM–EDS). Dried powders were mounted on conductive carbon tape and sputter−coated with gold before analysis. Elemental mapping was used to examine the spatial distributions of Fe, Mg, and O within the composite.

Transmission electron microscopy

Transmission electron microscopy and high−resolution transmission electron microscopy (TEM/HRTEM) were performed using a Talos F200X microscope (FEI, USA). Samples were ultrasonically dispersed in absolute ethanol and deposited onto copper grids supported with an ultrathin carbon film. TEM and HRTEM images were

used to examine the core−shell morphology and interfacial lattice fringes of nZVI@$Mg(OH)_2$.

Raman spectroscopy

Raman spectra were collected using a LabRAM HR Evolution confocal Raman spectrometer with a 532 nm excitation laser, using a 50× objective lens and a laser power of ~1 mW to avoid sample oxidation. Spectra were recorded over the range of 100−4000 cm^{-1}, and the Raman shift was calibrated using a single−crystal Si wafer before measurement. For clarity, Fig. 7b of the main manuscript displays the 100−1600 cm^{-1} region, which contains the diagnostic bands used for the phase−evolution discussion.

N_2 adsorption−desorption measurements

N_2 adsorption−desorption measurements were conducted at 77 K using a Micromeritics ASAP 2460 surface−area and porosity analyzer (USA). Samples were vacuum−degassed at 120 °C for 8 h before analysis. The specific surface area was determined using the Brunauer−Emmett−Teller (BET) method from the adsorption branch (P/P_0 = 0.05−0.30), and pore−size distributions were calculated using the Barrett−Joyner−Halenda (BJH) method. The total pore volume was determined at a relative pressure close to $P/P_0 \approx 0.99$.

Electrochemical measurements

Catalyst ink was prepared by dispersing 10.0 mg of material and 15 μL of 5 wt% Nafion solution in a mixture containing 1.0 mL of isopropanol and 0.5 mL of ultrapure water, followed by ultrasonication for 30 min. An aliquot of the homogeneous suspension was drop−cast onto a polished glassy carbon electrode (GCE, diameter = 3 mm), which was subsequently vacuum−dried at 60 °C for 8 h.

Electrochemical measurements were performed using a CHI660E electrochemical workstation in a conventional three−electrode configuration containing 0.1 mol·L^{-1} Na_2SO_4 as the supporting electrolyte, an Ag/AgCl reference electrode, and a Pt−wire counter electrode. Cyclic voltammetry (CV) was conducted over the potential range from −1.0 to +1.0 V at a scan rate of 50 mV·s^{-1}. Electrochemical impedance spectroscopy (EIS) was recorded at the stabilized open−circuit potential over a frequency range of 10^5–10^{-1} Hz using a sinusoidal perturbation amplitude of 5 mV.

For in situ open−circuit potential (OCP) measurements, the working electrode was first equilibrated in blank electrolyte until a stable baseline was reached (approximately 600 s). A concentrated perchlorate solution was then introduced to achieve a final ClO_4^- concentration of 10 mg·L^{-1}, and the OCP response was continuously recorded until 1200 s.

The complex impedance spectra were fitted using equivalent electrical circuits composed of solution resistance (R_s), resistance elements (R_i), and constant−phase elements (CPE_s). Bare nZVI was fitted using R_s − (R_1//CPE_1), whereas nZVI@$Mg(OH)_2$ required two serially connected relaxation elements and was fitted using R_s − (R_1//CPE_1) − (R_2//CPE_2). The CPE exponent P describes deviation from ideal capacitive behavior, with P = 1 corresponding to an ideal capacitor. Complex fitting with modulus weighting was used. Fitting quality was evaluated from χ^2, the weighted sum of squares, and the relative errors of the fitted parameters. Because two interfacial relaxation processes were resolved for nZVI@$Mg(OH)_2$, the total polarization resistance was calculated as $R_p = R_1+R_2$; for bare nZVI, $R_p = R_1$.

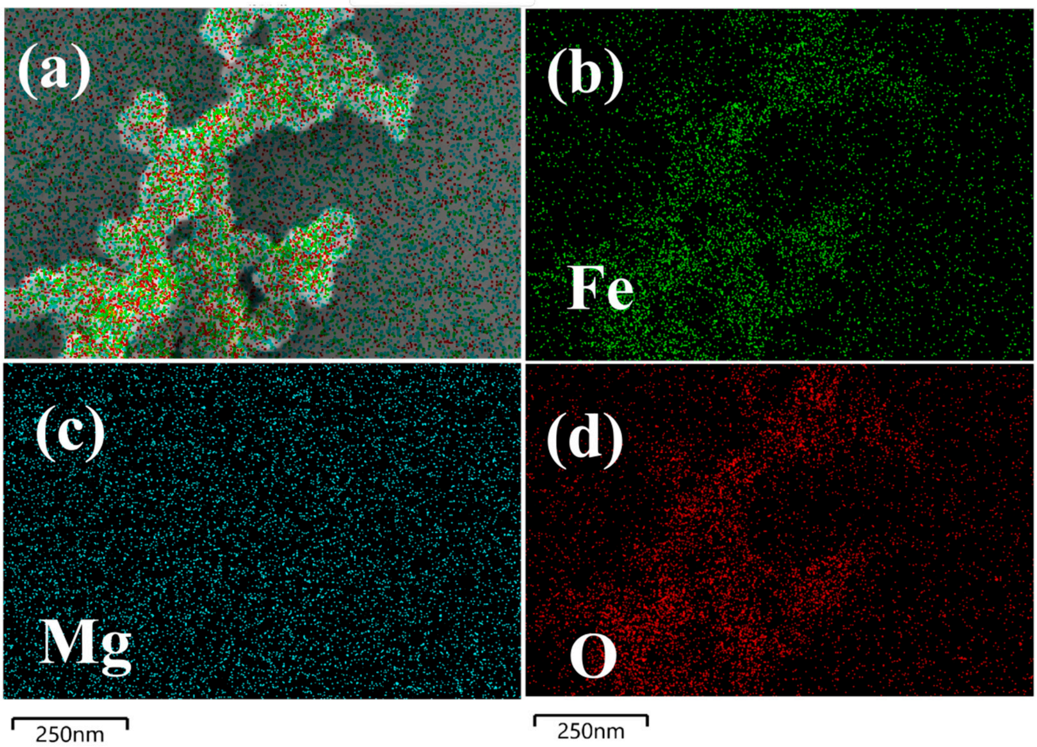

Fig. S1. Morphology and elemental distribution of nZVI@Mg(OH)$_2$: (a) electron microscopy image overlaid with Fe, Mg, and O elemental maps; individual elemental distributions of (b) Fe, (c) Mg, and (d) O.

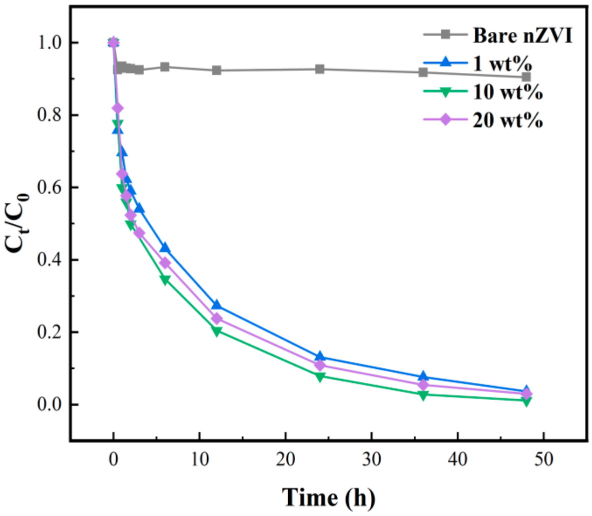

Fig. S2. Effect of Mg(OH)$_2$ loading on perchlorate removal by bare nZVI and nZVI@Mg(OH)$_2$ composites with Mg(OH)$_2$/nZVI mass ratios of 1, 10, and 20 wt%. Reaction conditions: material dosage = 4.35 g·L^{-1}, initial ClO_4^-

concentration = 10 mg·L^{-1}, initial pH = 4.0, T = 313 K, and reaction time = 48 h. The composite containing 10 wt% $Mg(OH)_2$ exhibited the highest ClO_4^- removal efficiency (98.37%) and was selected for subsequent experiments. Data are presented as mean ± SD from three parallel experiments.

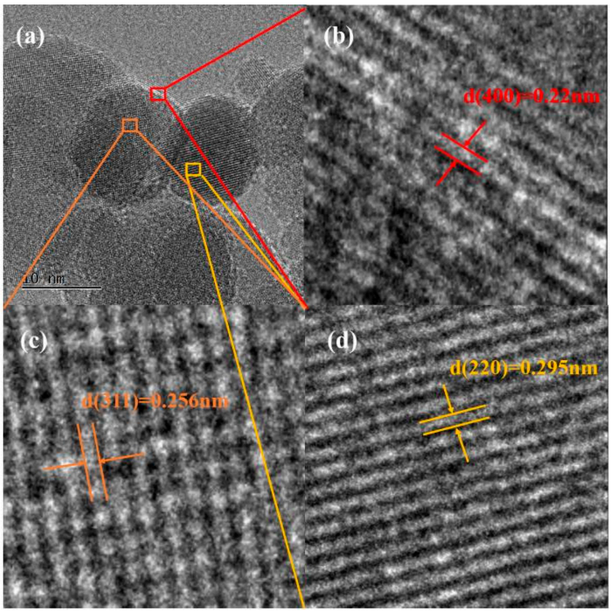

Fig. S3. (a) TEM image of nZVI@$Mg(OH)_2$; (b−d) HRTEM images of nZVI@$Mg(OH)_2$ after perchlorate reaction

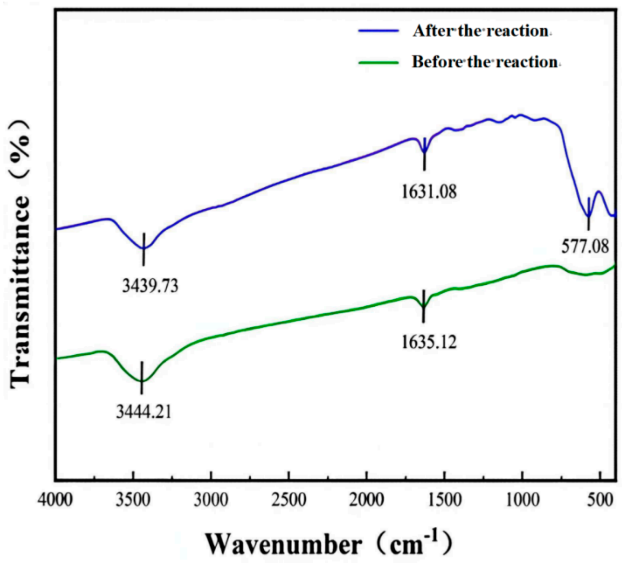

Fig. S4. FTIR spectra of nZVI@Mg(OH)$_2$ before and after reaction with perchlorate

Table S1. Specific surface area and pore−structure parameters of bare nZVI and $nZVI@Mg(OH)_2$.

Sample	BET specific surface area ($m^2\cdot g^{-1}$)	Average pore diameter (nm)	Total pore volume ($cm^3\cdot g^{-1}$)
Bare nZVI	4.57	21.86	0.0242
$nZVI@Mg(OH)_2$	67.29	15.11	0.2542

Table S2. BET specific surface area and 48 h ClO_4^- removal efficiency between B,N−doped porous−carbon−supported nZVI and $nZVI@Mg(OH)_2$.

Sample	BET specific surface area ($m^2\cdot g^{-1}$)	48 h ClO_4^- removal
B,N−doped porous−carbon−supported nZVI	65.37	59.91%
$nZVI@Mg(OH)_2$	67.29	98.37%

Note: B,N−doped porous−carbon−supported nZVI was prepared using a two−step procedure. First, a carbon precursor was mixed with urea and a boron source, dried at 80 °C, and pyrolyzed under an N_2 atmosphere at 700 °C for 4 h to obtain B,N−doped porous carbon (B/N−CN). Subsequently, B/N−CN was dispersed in an $FeCl_3$ solution, followed by the dropwise addition of $NaBH_4$ under continuous stirring to reduce Fe^{3+} to Fe^0 and immobilize nZVI on the porous carbon surface. The resulting composite was collected by centrifugation and washed with deionized water and ethanol. The ClO_4^- removal experiments for B,N−doped porous−carbon−supported nZVI and $nZVI@Mg(OH)_2$ were conducted under identical reaction conditions, with the same effective Fe^0 concentration in both systems.

Table S3. Pseudo−first−order kinetic parameters for ClO_4^- removal by $nZVI@Mg(OH)_2$ at different material dosages.

Material dosage (g)	k_{obs} (h^{-1})	$t_{1/2}$ (h)	R^2
0.05	0.031	22.36	0.993

0.075	0.049	14.15	0.987
0.1	0.059	11.75	0.981
0.125	0.069	10.05	0.984
0.15	0.075	9.24	0.992

Note: Initial ClO_4^- concentration = 10 mg·L^{-1}, initial pH = 4.0, and T = 313 K.

Table S4. Pseudo−first−order kinetic parameters for ClO_4^- removal by nZVI@Mg(OH)$_2$ at different initial ClO_4^- concentrations.

Initial ClO_4^- concentration (mg·L^{-1})	k_{obs} (h^{-1})	$t_{1/2}$ (h)	R^2
5	0.084	8.25	0.993
10	0.083	8.35	0.999
50	0.059	11.75	0.983
100	0.058	11.95	0.984

Note: Material dosage = 4.35 g·L^{-1}, initial pH = 4.0, and T = 313 K.

Table S5. Pseudo−first−order kinetic parameters for ClO_4^- removal by nZVI@Mg(OH)$_2$ at different initial pH values.

Nominal initial pH	Measured initial pH	pH at 48 h	k_{obs} (h^{-1})	$t_{1/2}$ (h)	R^2
2	1.80	7.02	0.019	36.48	0.987
4	4.00	8.28	0.067	10.34	0.967
6	6.12	8.17	0.068	10.19	0.978
8	7.88	8.30	0.063	11.00	0.991
10	9.96	8.26	0.057	12.16	0.988

Note: Material dosage = 4.35 g·L^{-1}, initial ClO_4^- concentration=10 mg·L^{-1}, and T = 313 K.

Table S6. Effects of coexisting anions on perchlorate removal by nZVI@$Mg(OH)_2$.

Ion	Concentration (mg/L)	ClO_4^- removal at 48 h (%)
Cl^-	10	93.58 ± 1.44
Cl^-	200	88.13 ± 1.38
Cl^-	500	72.38 ± 1.22
Cl^-	1000	56.56 ± 1.07
NO_3^-	10	78.66 ± 1.29
NO_3^-	200	56.72 ± 1.07
NO_3^-	500	7.49 ± 0.57
NO_3^-	1000	1.59 ± 0.52
SO_4^{2-}	10	47.42 ± 0.97
SO_4^{2-}	200	30.32 ± 0.80
SO_4^{2-}	500	9.51 ± 0.57
SO_4^{2-}	1000	7.00 ± 1.20

Note: Under the same benchmark conditions but without added coexisting anions, the 48 h ClO_4^- removal efficiency was 98.37%. This value is shown as the horizontal dashed reference line in Fig. 5d of the main manuscript. Data are presented as mean ± SD (n = 3).

Table S7. Pseudo−first−order kinetic parameters for ClO_4^- removal by nZVI@$Mg(OH)_2$ at different reaction temperatures.

Temperature (°C)	k_{obs} (h^{-1})	$t_{1/2}$ (h)	R^2
20	0.021	33.01	0.869
30	0.034	20.39	0.968
40	0.061	11.36	0.988
50	0.153	4.53	0.958

Note: Material dosage=4.35 g·L^{-1}, initial ClO_4^- concentration=10 mg·L^{-1}, and initial pH=4.0. Arrhenius regression parameters: linear regression equation is ln k = −6129.31471×(1/T)+16.9213; slope is −6129.31471 K; standard error of the slop is ±726.07325 K; intercept is 16.9213 ±2.35952; R^2=0.9727; E_a=50.96±6.04 kJ·mol^{-1}.

Note for Tables S3–S5 and S7. The single−factor experiments summarized in Tables S3–S5, and S7 were conducted independently using separately synthesized batches of nZVI@$Mg(OH)_2$. Accordingly, kinetic comparisons were made within each experimental series. The reported k_{obs} values were derived from the decrease in aqueous ClO_4^- concentration and therefore represent apparent disappearance/removal kinetics rather than product−resolved reduction rates. All batch experiments were performed in triplicate (n = 3), and experimental results are reported as mean ± SD where applicable.

Table S8. Equivalent−circuit fitting parameters for EIS spectra of bare nZVI and nZVI@$Mg(OH)_2$.

Fitting parameters	Bare nZVI	nZVI@$Mg(OH)_2$
Equivalent circuit	$R_s - (R_1//CPE_1)$	$R_s - (R_1//CPE_1) - (R_2//CPE_2)$
R_s (Ω)	27.48	26.03
R_1 (Ω)	15209	78.66
CPE1-T	1.31×10^{-4}	4.11×10^{-4}
CPE1-P	0.818	0.701
R_2 (Ω)	/	10699
CPE2-T	/	2.83×10^{-4}
CPE2-P	/	0.804
Total fitted R_P (kΩ)	15.209	10.778
χ^2	0.00486	0.00197
Relative error of resistance parameters	2.86% (R_1)	11.37% (R_1); 5.29% (R_2)

Note: R_p denotes the total fitted polarization resistance. For bare nZVI, R_p = R_1; for nZVI@$Mg(OH)_2$, $R_p = R_1+R_2$. The use of R_p avoids direct comparison of individual resistance elements between equivalent circuits containing different numbers of relaxation processes.

Table S9. Fe 2p XPS fitting results for nZVI@$Mg(OH)_2$ before and after perchlorate reaction.

Fe species	Binding energy before reaction (eV)	Relative abundance before reaction (%)	Binding energy after reaction (eV)	Relative abundance after reaction (%)
Fe^0	706.98	4.95	–	–
Fe(II)	710.31	60.13	710.25	34.10
Fe(III)	712.43	34.92	711.96	65.90

Table S10. O 1s XPS fitting results for nZVI@$Mg(OH)_2$ before and after perchlorate reaction.

O species	Binding energy before reaction (eV)	Relative abundance before reaction (%)	Binding energy after reaction (eV)	Relative abundance after reaction (%)
Lattice O^{2-}	529.76	53.05	529.71	48.67
Hydroxyl O	531.29	34.83	531.01	40.20
Adsorbed H_2O	532.93	12.12	532.59	11.13
